# Interaction of two antitumor peptides with membrane lipids – Influence of phosphatidylserine and cholesterol on specificity for melanoma cells

**DOI:** 10.1371/journal.pone.0211187

**Published:** 2019-01-25

**Authors:** Christina Wodlej, Sabrina Riedl, Beate Rinner, Regina Leber, Carina Drechsler, Dennis R. Voelker, Jae-Yeon Choi, Karl Lohner, Dagmar Zweytick

**Affiliations:** 1 Institute of Molecular Biosciences, University of Graz, Graz, Austria; 2 BioTechMed-Graz, Graz, Austria; 3 Center for Medical Research, Medical University of Graz, Graz, Austria; 4 BIOSS and Institute of Pharmaceutical Sciences, University of Freiburg, Freiburg i. Br., Germany; 5 Department of Medicine, National Jewish Health, Denver CO, United States of America; Oregon State University, UNITED STATES

## Abstract

R-DIM-P-LF11-322 and DIM-LF11-318, derived from the cationic human host defense peptide lactoferricin show antitumor activity against human melanoma. While R-DIM-P-LF11-322 interacts specifically with cancer cells, the non-specific DIM-LF11-318 exhibits as well activity against non-neoplastic cells. Recently we have shown that cancer cells expose the negatively charged lipid phosphatidylserine (PS) in the outer leaflet of the plasma membrane, while non-cancer cells just expose zwitterionic or neutral lipids, such as phosphatidylcholine (PC) or cholesterol. Calorimetric and zeta potential studies with R-DIM-P-LF11-322 and cancer-mimetic liposomes composed of PS, PC and cholesterol indicate that the cancer-specific peptide interacts specifically with PS. Cholesterol, however, reduces the effectiveness of the peptide. The non-specific DIM-LF11-318 interacts with PC and PS. Cholesterol does not affect its interaction. The dependence of activity of R-DIM-P-LF11-322 on the presence of exposed PS was also confirmed *in vitro* upon PS depletion of the outer leaflet of cancer cells by the enzyme PS-decarboxylase. Further corresponding to model studies, cholesterol depleted melanoma plasma membranes showed increased sensitivity to R-DIM-P-LF11-322, whereas activity of DIM-LF11-318 was unaffected. Microscopic studies using giant unilamellar vesicles and melanoma cells revealed strong changes in lateral distribution and domain formation of lipids upon addition of both peptides. Whereas R-DIM-P-LF11-322 enters the cancer cell specifically via PS and reaches an intracellular organelle, the Golgi, inducing mitochondrial swelling and apoptosis, DIM-LF11-318 kills rapidly and non-specifically by lysis of the plasma membrane. In conclusion, the specific interaction of R-DIM-P-LF11-322 with PS and sensitivity to cholesterol seem to modulate its specificity for cancer membranes.

## Introduction

Cancer is one leading cause of death with 9.6 million related deaths in 2018 (http://www.who.int/en/news-room/fact-sheets/detail/cancer) [1]. Despite enormous progress in therapy over the last decades, there are still many types of cancer that exhibit poor treatability or require therapies provoking side effects. One form of cancer with poor prognosis is malignant melanoma with a median survival rate of only six months [2]. It is the most dangerous form of skin cancer causing 80% of related deaths and the cancer with the strongest increase of incidences at present [3]. So far, the only FDA approved agents for treatment of metastatic melanoma are cytostatic DTIC and immunotherapeutic Interleukin-2 (IL-2), ipilimumab, an anti-CTLA4-antibody and nivolumab, which blocks the programmed cell death protein 1 (PD-1) of T-cells. Median progression-free survival is 11.5 months for nivolumab plus ipilimumab as compared with 6.9 months for nivolumab alone [4]. Further, two BRAF targeting inhibitors are vemurafenib and dabrafenib. The problem of BRAF kinase inhibitors is potential development of resistance within 6 to 7 months [5,6]. Due to severe side effects and main dependence on mutations in the cancer cells, lack of response or even formation of resistance upon therapy, the severe need for new targets on melanoma is reasonable.

In this respect it is of great interest that Utsugi et al. [7] reported in 1991 that tumorigenic cells, such as melanoma, express higher levels of the negatively charged phospholipid phosphatidylserine (PS) than non-neoplastic cells, such as keratinocytes. PS exposure during malignant transformation was also reported by Zwaal et al. [8], Ran et al. [9,10] and others [11–14]. Recently, we were able to show that not only tumor cell lines of melanoma but also primary cultures and metastases thereof, besides other cancer types expose the negatively charged phospholipid PS [15]. Normally the outer leaflet of the plasma membrane only consists of neutral phospholipids like phosphatidylcholine (PC) and sphingomyelin (SM). Only the inner leaflet comprises the negatively charged phospholipid PS [16]. Oxidative stress, acidity, thrombin, and inflammatory cytokines are reported to induce exposure of anionic phospholipids, such as PS, to the outer leaflet and are therefore discussed to cause a loss of asymmetry during malignant transformation [10], which is normally controlled by active ATP-dependent phospholipid translocases and an inactive Ca^2+^-dependent scramblase [16]. Cells, exposing PS, should actually lead to recognition by macrophages and induction of cell death. Surprisingly, cancer cells prevent such detection by macrophages and normally induced apoptosis. E.g., melanoma inhibit the expression of a gene encoding APAF1, the apoptotic protease activating factor-1 [17,18]. Nevertheless, the exposure of the negatively charged PS by cancer cells might be an Achilles’ heel of cancer providing a potential target for cationic molecules which can discriminate between neutral surfaces of non-cancer and negatively charged surfaces of cancer membranes.

One strategy on which the innate immune system relies as defense against microorganisms and tumor cells is the use of antimicrobial/antitumor peptides (AMPs, ACPs) as a part of the first line defense. AMPs are wide spread in nature, especially in the plant and animal kingdoms [19,20]. The cationic peptides are discussed to preferentially interact with negatively charged phospholipids via electrostatic interaction [21]. In addition to the antimicrobial effects, more and more studies report of anticancer activity exhibited by some AMPs. APD (the antimicrobial peptide database) lists 212 anticancer/antitumor peptides so far, as for example magainin from the African clawed frog *Xenopus laevis*, tachyplesin from the horseshoe crab or human as well as bovine and human lactoferricin (LFcin) [22–24]. Zweytick et al. [25] reported that variants of a peptide comprising the membrane active region amino acids 21 to 31 of LFcin, the so called peptide LF11 (FQWQRNIRKVR-NH_2_), exhibited increased antimicrobial activities against several gram-positive and -negative bacterial strains [25–27]. These peptides showed correlation of bacterial activity with perturbation of bacterial model liposomes composed of the negatively charged lipids phosphatidylglycerol or cardiolipin. Amongst the potent non-acylated peptides designed in this study were, for instance, LF11-322 (PFWRIRIRR-NH2, +5 net charge) or LF11-318 (FWQRRIRRWRR-NH2, +7 net charge) [26,27].

Due to the fact that cancer like bacterial cells expose a negatively charged lipid, namely phosphatidylserine (PS), Riedl et al. [15] tested if LF11-322 and other antibacterial active LF11-derivatives also reveal specific killing efficiency against cancer cells. However, anti-cancer activity of the short peptides was low [28]. Since Yang et al. [29] reported that for potent antitumor activity a minimum positive net charge (at least + 7) and hydrophobicity is important, di-(retro-) peptides were designed by addition of the same or its retro sequence to reach such a minimum net charge, such as R-DIM-P-LF11-322, which is LF11-322 linked to its retro sequence via a proline residue. It turned out to fulfill all important parameters: a minimum length, a high positive net charge, a high affinity to the target PS and an adequate hydrophobicity (PFWRIRIRR-P-RRIRIRWFP-NH2, + 9 net charge) [28]. Most importantly, a highly increased specific activity against melanoma cell lines (A375; SBcl-2) and melanoma metastases (WM164) [28] over non-cancer cells as normal dermal fibroblasts (NHDF) was shown compared to low specific activity of the short peptide LF11-322 (R-DIM-P-LF11-322: LC_50; A375_ 9,5 μM, LC_50; NHDF_ 65 μM; LF11-322: LC_50; SbCl2_ >80 μM, LC_50; NHDF_ >80 μM). Activity was further increased 10 fold compared to its parent peptide LFcin [30]. The di-peptide of the antibacterial active peptide LF11-318, DIM-LF11-318 also revealed to be highly antitumor active but was non-selective (FWQRRIRRWRR-FWQRRIRRWRR-NH2, + 13 net charge) with a LC_50_ of 4 μM for A375 and 2 μM for NHDF [30]. As shown by Riedl et al. [30], the online program PEP-FOLD [31–34] for in silico secondary structure prediction revealed a conformation of two β-strands for R-DIM-P-LF11-322 and an α-helical structure without a loop for DIM-LF11-318. Within the study further peptide variants with non-looped α-helical structures exhibited non-selective toxicity for cancer and non-cancer cells, whereas looped structures could be correlated to highly cancer specific peptides. Specific peptides were shown to kill cancer cells slowly through induction of apoptosis in a concentration dependent manner; whereas non-specific peptides killed cancer cells fast via necrosis.

Besides phospholipids the plasma membrane of eukaryotic cells also comprises the neutral lipid cholesterol (up to one molecule cholesterol per phospholipid [35]). Cholesterol is reported to preferentially interact with sphingolipids in so called rafts [36,37]. Matsuzaki et al. [38] reported that cholesterol depletion of erythrocytes led to increased sensitivity against the AMP magainin, suggesting that cholesterol may have a protective role against AMPs.

Based on work in our lab by Riedl et al. [15,17,28,30,39] the aim of the present study was to analyze differences in the mode of action and specificity of two human lactoferricin derivatives on malignant melanoma cells, namely the cancer specific R-DIM-P-LF11-322 and the cancer active, but non-specific DIM-LF11-318. The main focus lays on effects of the lipids PS, PC and cholesterol on the peptide interaction with its target membrane. Therefore, lipid model systems mimicking cancer cells with different ratios of PS, PC and cholesterol in absence and presence of either R-DIM-P-LF11-322 or DIM-LF11-318 were analyzed with different biophysical methods. Furthermore, the peptide effects *in vitro* on malignant melanoma cells in absence of plasma membrane cholesterol or PS in the outer leaflet as well as changes in morphology and lipids of certain cell organelles were determined. To deplete plasma membrane PS PS-decarboxylase from *Plasmodium knowlesi* (PkPSD) was used, which has been identified and characterized by its ability to complement a yeast mutant defective in *de novo* synthesis of PE (*psd1Δpsd2Δdpl1Δ* strain) [40]. Deletion of N-terminus membrane domain of the protein and MBP fusion greatly improved the solubility of the enzyme [40–42].

Our results will help to further optimize the peptides with the purpose to design a new, specific and potent therapy against malignant melanoma. The LFcin derived antitumor peptides are part of patent publications EP2943215 B1 “Lactoferricin derived peptides for use in the treatment of cancer” and US9872891B2 “Peptides for the Treatment of Cancer”; current assignee: NEWFIELD THERAPEUTICS CORPORATION.

## Materials and methods

### Lipids

The lipids 1,2-diplamitoyl-*sn*-glycero-3-phosphocholine (DPPC), 1,2-dipalmitoyl-*sn*-glycero-3-phospho-L-serine (sodium salt) (DPPS), 1-palmitoyl-2-oleoyl-*sn*-glycero-3-phosphocholine (POPC), 1-palmitoyl-2-oleoyl-*sn*-glycero-3-phospho-L-serine (sodium salt) (POPS) and fluorescently labeled 1,2-dioleoyl-*sn*-glycero-3-phosphoethanolamine-N-(lissamine rhodamine B sulfonyl) (ammonium salt) (RhoDOPE) were purchased from Avanti Polar Lipids, Inc. (Alabaster, AL, USA). Cholesterol was purchased from Sigma-Aldrich Co. LLC (Deisenhofen, Germany). Lipids were used without any further purification. Stock solutions of DPPC and POPC were prepared in a mixture of chloroform and methanol of 2:1 (v/v). Stock solutions of DPPS and POPS were prepared in a mixture of chloroform and methanol of 9:1 (v/v). Cholesterol and RhoDOPE were dissolved in pure chloroform. Stock solutions were stored at– 20°C. Purity of purchased lipids was >99%.

### Peptides

The C-terminally amidated peptides R-DIM-P-LF11-322 (PFWRIRIRR-P-RRIRIRWFP-NH2, M = 2677.4 g/mol), DIM-LF11-318 (FWQRRIRRWRR-FWQRRIRRWRR-NH2, M = 3413.1 g/mol) and the fluorescently labeled peptide (5–6)-FAM-R-DIM-P-LF11-322 ((5–6)-FAM-PFWRIRIRR-P-RRIRIRWFP-NH2, M = 3035.7 g/mol) were purchased from PolyPeptide Group (San Diego, CA, USA). A purity of >95% for all peptides was determined by RP-HPLC. Stock solutions of R-DIM-P-LF11-322 and DIM-LF11-318 were prepared in acetic acid (0.1% v/v) at a concentration of about 3 mg/ml and treated with ultrasonication for better solubility. The peptide concentrations of the non-labeled peptides were determined via measurement of UV-absorbance of tryptophan at 280 nm (using NanoDrop ND 1000 (Peqlab, VWR International, Inc. Erlangen, Germany)). The fluorescently labeled peptide was dissolved in Dulbecco’s Phosphate Buffered Saline (DPBS, Gibco) at a concentration of about 3 mg/ml. Peptide solutions were stored at 4°C.

### Model system studies

#### Liposome preparation

Liposomes were prepared as described previously [28]. Briefly, 1 mg of respective phospholipid stock solution was dried under a stream of nitrogen and stored in vacuum overnight to completely remove organic solvents. The dry lipid film was then dispersed in phosphate buffered saline (PBS, 20 mM NaPi, 130 mM NaCl, pH 7.4) and hydrated at a temperature well above the gel to fluid phase transition of the respective phospholipid under intermittent vigorous vortex-mixing. The lipid concentration was 0.1 weight% for calorimetric experiments. Hydration was carried out in presence or absence of peptides at a lipid-to-peptide molar ratio of 25:1 using protocols for DPPC (hydration at 50°C for two hours, vortexing every 15 min) and DPPS (hydration at 65°C for two hours, vortexing every 15 min). The hydration of molar ratios of lipid mixtures DPPC/DPPS 1:1 and 3:1 as well as DPPC/DPPS/Cholesterol (1:1:0.25 and 1:1:0.5) were performed at 65°C for 2 hours by intermittent vortexing partially accompanied by one freeze-thaw cycle every 15 minutes. All liposomal mixtures for calorimetric experiments were also hydrated without the freeze and thaw procedure. The fully hydrated samples were stored for at least 1 hour at room temperature until measurement.

For the hydration of liposomes for zeta potential and size measurement, as well as circular dichroism studies, the peptides were not added during the preparation procedure. For these experiments liposomes with lipid concentrations of 1 weight% were extruded after preparation ten times through a 200 nm Whatman Nuclepore Track-Etched Membrane (Sigma Aldrich Co. LLC), using a Mini-Extruder of Avanti Polar Lipids Inc.. Peptides were added afterwards at a lipid-to-peptide molar ratio of 25:1. Before the measurement the liposomal preparations was diluted to 0.0005 weight%

#### Differential scanning calorimetry (DSC)

Preparation of respective liposomes for DSC experiments is described above. 0.1 weight% of lipid was hydrated in absence or presence of peptides at a molar ratio of lipid-to-peptide of 25:1. DSC measurements were performed with a differential scanning calorimeter (VP-DSC) from MicroCal, Inc. (Northhampton, MA, USA). Heating scans were performed at a scan rate of 30°C/h (pre-scan thermostating 30 min) with a final temperature of approximately 10°C above the main transition temperature (T_m_) and cooling scans at the same scan rate (pre-scan thermostating 1 min) with a final temperature approximately 20°C below T_m_. The heating/cooling cycle was performed two times. For the mixtures the first two cycles occurred between 25°C and 65°C and the second two cycles occurred between 25°C and 70°C. For analyses of the characteristics of the respective thermotropic phase behaviors the MicroCal Origin Software (VP-DSC version) was used. Briefly the peak areas were integrated after normalization to the phospholipid concentration and baseline adjustment for calculation of the enthalpies of the pre-transition and the main transition, ΔH_pre_ and ΔH_m_, respectively. The phase transition temperatures of the pre- and the main transition (T_pre_ and T_m_) were determined as the maxima of the peaks. The T_1/2_ (half-width) was defined as peak width at half height [43]. Deconvolution was used for analyses of data of overlapping phase transitions.

#### Zeta potential and size

Preparation of respective liposomes for Zeta potential and size experiments is described above. The lipid concentration was 0.0005 weight%. Peptides were added at a molar ratio of lipid-to-peptide of 25:1 to already hydrated and extruded liposomes. Zeta potentials of liposomal mimics of cancer and non-cancer membranes in absence or presence of peptides were determined with a Zetasizer Nano ZSP (Malvern Instruments, Herrenberg, Germany) by determining the electrophoretic mobility and applying the Henry equation. The zeta potential was calculated according to the Smoluchovski model used for aqueous solutions. Furthermore, the size of the particles was measured by dynamic light scattering (DLS).

Freshly extruded liposomes were diluted with HEPES cesium chloride buffer (10 mM HEPES, 2 mM CsCl, pH 7.1) to a final lipid concentration of 0.05 mg/ml. Peptides and Ca^2+^ were added to the liposomal suspension at a lipid-to-peptide molar ratio of 25:1 and a final concentration of 1 mM, respectively. Three measurements were performed after 5 min of incubation at 25°C (120 seconds equilibration time) in Cell DTS1070 disposable folded capillary cells (Malvern) with 10–30 runs and 60 s delay between measurements. Attenuation and voltage selection was automatic, analysis was auto-mode. Size was measured at 25°C after each zeta potential measurement. 173° backscatter was used as angle for detection.

In case the Pdi (polydispersity index) was lower than 0.5 the z-average (mean value of the size; calculated from the signal intensity of light scattering measurement) was used for comparison of sizes. When the Pdi was above 0.5 the number mean values were used being of better validity.

#### Giant unilamellar vesicles (GUVs)

Giant unilamellar vesicles (GUVs) were prepared by electroformation [44] following the protocol kindly provided by Karin A. Riske (Departamento de Biofísica, Universidade Federal de São Paulo, São Paulo, Brazil). Lipid mixtures composed of molar ratios of POPC/DPPS 1:1 and 3:1, DPPC/POPS 1:1 and 3:1 and 1% (w/v) RhoDOPE were used. Peptides were added to the GUVs at a concentration of 50 μM (approximate lipid-to-peptide molar ratio of 3:1) and incubated for 30–60 min at room temperature in the dark. The molar mixtures DPPC/POPS/Cholesterol 1:1:0.25 and 1:1:0.5 had to be analyzed immediately after peptide addition due to fast destruction in presence of peptides. Fluorescence imaging was performed in Ibidi μ-slides 18 wells with excitation wavelength of 558 nm and emission wavelength of 582 nm.

#### Circular dichroism (CD)

Measurements were performed on a Jasco J 715 Spectropolarimeter (Jasco, Gross-Umstadt, Germany) at room temperature using quartz cuvettes with an optical path length of 0.02 cm. The CD spectra were measured between 260 nm and 180 nm with a 0.2 nm step resolution. To improve accuracy 3 scans were averaged. Peptide was dissolved in 10 mM Hepes (pH 7.4) to a final concentration of 200 μM. Spectra were measured in the absence and presence of LUVs of 20 mM POPS, 20 mM POPS/Cholesterol (3:1; molar ratio) (Avanti Polar Lipids, Alabaster, USA) mimicking cancer mammalian membranes. The respective lipid-to-peptide molar ratio was 25:1. Background signals were abstracted after measurements. Percentage secondary structure calculations were done using Dichroweb, Contin-LL (Provencher & Glockner Method) Convolution Program using reference set 7 [45,46].

### *In vitro* studies

#### Cell lines and cultures

Normal human dermal fibroblasts (NHDF) and melanoma cells A375 were purchased from PromoCell, Inc. (Heidelberg, Germany) and ATCC (American Type Culture Collection, Manassas, VA, USA), respectively. A375 cells were cultured in Dulbecco’s Modified Eagle Medium with GlutaMAX, high glucose and phenol red (DMEM, Gibco) supplemented with 10% fetal bovine serum (FBS, Gibco). NHDF cells were cultured in Fibroblast Growth Medium 2 (PromoCell, Inc., Heidelberg, Germany). Cells were kept at 37°C and 5% CO_2_.

At 90% confluence cells were passaged with accutase (Gibco, Thermo Fisher Scientific, USA). All cell cultures were periodically checked for mycoplasma.

#### Fluorescence microscopy

Experiments were performed with a Leica DMI6000 B with IMC using a Leica DFC360 FX camera and AF 6000 software (Leica Microsystems, Vienna, Austria). Peptides were added at concentrations of 10 μM, if not elevated otherwise.

#### Cell culturing

For microscopic experiments, aliquots of 10^4^ to 2*10^4^ cells/300 μl were seeded on Ibidi μ-Slide 8 wells (Martinsried, Munich, Germany) and cultured for up to three days to gain a confluent layer (app. 5*10^4^ to 10^5^ cells/well).

#### Fixation of cells

Fixation of cells was performed before (cholesterol and mitochondria) or after (Golgi) staining of the cells. For fixation, cells were washed gently with Dulbecco's phosphate-buffered saline without calcium and magnesium (DPBS, Gibco) and fixed with 1% (w/v) paraformaldehyde for 10 min at RT.

#### Propidium iodide (PI)-uptake

PI staining (excitation wavelength 538 nm; emission wavelength 617 nm) was used to visualize the toxicity of peptides (10 μM) indicated by binding of PI to DNA. 2 μl of a 50 μg/μl PI-solution (Biosource, Camarillo, CA, USA) were added to each well and incubated for 5 min in the dark.

#### Nuclear staining

NucBlue Live ReadyProbes Reagent (Molecular Probes Inc., Eugene, OR, USA) (excitation wavelength 359 nm; emission wavelength 461 nm) was used for co-staining of nucleus with mitochondria or Golgi. One drop of the ready to use kit (~ 5 μl) was transferred into each well and incubated for 5 minutes before microscopic analyses were performed.

#### Cholesterol staining

Filipin complex (Sigma-Aldrich Co. LLC) (excitation wavelength 352 nm; emission wavelength 454 nm) was used for cholesterol staining of cell membranes of NHDF and A375. Filipin was solved in DMSO at a concentration of 25 mg/ml. After fixation, cells were stained with filipin (0.05 mg/ml) for at least 10 minutes at 37°C. Cells were washed and PI was added. For experiments in presence of peptides (labeled and unlabeled), peptides were added to confluent cells and incubated for 0, 30, 60 and 120 min at 37°C prior to fixation, cholesterol and PI staining of DNA. Filipin staining was also used for cholesterol depletion control experiments.

#### Mitochondrial staining

MitoTracker Deep Red (Molecular Probes Inc., Eugene, OR, USA) (excitation wavelength 650 nm; emission wavelength 668 nm) was used for staining of mitochondria in A375 cells. The stock solution (1 mM in DMSO) was diluted 1:20000 in DPBS. At 90% confluence cells were fixed and stained with 0.05 μM MitoTracker Deep Red for 15 minutes at 37°C. For experiments in presence of peptides (labeled and unlabeled), peptides were added to confluent cells and incubated for 0, 30, 60 and 120 min prior to fixation, mitochondrial and nuclear staining.

#### Golgi staining

The staining of the Golgi apparatus (Golgi) with BODIPY TR Ceramide complexed to BSA (Molecular Probes Inc., Eugene, OR, USA) (excitation wavelength 589 nm; emission wavelength 617 nm) was performed following the manufacturer’s protocol. Briefly, cells were incubated with peptides for the desired period of time (0 min, 30 min, 60 min and 120 min) prior to the Golgi staining. Then, cells were washed twice with DPBS and incubated with 5 μM working solution for 30 min at 4°C. Cells were washed twice with DPBS (4°C) and again incubated for 30 min with fresh medium at 37°C. Cells were washed again with pre-warmed medium and fixed with 1% (w/v) paraformaldehyde for 10 min at room temperature, nuclear staining followed.

#### Peptide localization studies

Melanoma cells (A375) were incubated for two hours with 10 μM fluorescently labeled ((5–6)-FAM-) R-DIM-P-LF11-322 (excitation wavelength 495 nm; emission wavelength 519 nm). Golgi staining was performed with BODIPY ceramide, mitochondrial staining with MitoTracker Deep Red and nuclear staining with NucBlue.

#### Cholesterol depletion studies-PI-uptake

Prior to cholesterol depletion studies with peptides different amounts of the cholesterol depletion agent methyl-β-cyclodextrin (MβCD) were tested to determine the minimal amount for the depletion studies. Briefly, cells were treated with 0.04, 1 and 5 mM MβCD in DPBS supplemented with 10% FBS for 30 min at 37°C followed by cholesterol staining as described above.

For cholesterol depletion experiments, cells were grown in 175 cm^2^ culture flasks to 90% confluence. Cells were treated with 0, 5 and 10 mM MβCD in DBPS supplemented with 10% FBS with gentle shaking at 100 rpm and 37°C. Afterwards cells were collected and aliquots of 10^5^ cells/100 μl DMEM (without phenol red) supplemented with 10% FBS were seeded into a dark 96 well plate. Different amounts of peptide (0–40 μM) and 2 μl PI (50 μg/μl) were added and PI-uptake was measured after 0, 1, 2, and 4 h using the GloMax Multi+ Detection System (Promega, Madison, WI, USA). Cytotoxicity was calculated from the percentage of PI positive cells in media alone (P_0_) and in the presence of peptide (P_X_). (see Eq 1). Triton-X-100 was used to determine 100% of PI positive cells (P_100_).

$\%PI-uptake=\frac{100*(P_{x}-P_{0})}{\left(P_{100}-P_{0}\right)}$**Eq 1**: Equation for PI-uptake calculation

#### Reduction of PS exposed by cancer cells by the enzyme PS-decarboxylase PSD

PS exposure of melanoma cells A375 was determined using the Annexin V-Alexa 488 apoptosis detection kit (Molecular Probes Inc., Eugene, OR, USA). Briefly, 10^5^ cells were pretreated with 1x (2.6 μg), 2x (5.2 μg) and 5x (13.0 μg) PkPSD in DMEM without FBS for 30 min at 37°C. Subsequently, cells were washed once with Annexin binding buffer and stained with 5 μl Annexin V-Alexa 488 and 2 μl PI (50 μg/μl) for 5 min at RT in the dark. Cells were washed again and fluorescence (Annexin V-Alexa Fluor 488, λ_ex_ = 488 nm and λ_em_ = 530 nm; PI, λ_ex_ = 536 nm and λ_em_ = 617 nm) was recorded immediately using the GloMax Multi+ Detection System (Promega, Madison, WI, USA). Untreated cells were taken as a control for 100%.

The effect of reduced PS levels of PkPSD-treated melanoma cells A375 on R-DIM-P-LF11-322 and DIM-LF11-318 cytotoxicity was measured by PI uptake. Cells were pretreated with PkPSD as mentioned above. Subsequently respective peptides at a concentration of 5 μM were added and the PI uptake was measured (λ_ex_ = 536 nm and λ_em_ = 617 nm) at different time points (0, 1, 2 and 4 hours) using the GloMax Multi+ Detection System (Promega, Madison, WI, USA). For the 100% value (P_100_) 2 μl Triton (10% v/v) were added after the time series and after two minutes of incubation the PI fluorescence in each well was measured. For peptide cytotoxicity calculation the percentage of PI positive cells was measured in presence of the peptides (P_x_) and of PI positive cells in media without any peptide (P_0_) (see Eq 1).

The enzyme PkPSD [40,42], was tested for its activity before use by detection of product phosphatidylethanolamine by thin layer chromatography [47].

### Statistical analysis

Values are presented as the mean ± SEM. Cell culture and microscopy studies were repeated at least three times. All studies were performed with defined stock solutions of lipids and peptides with purity higher than 99% and 96%, respectively. For microscopy studies, a set of data being representative of the respective results is shown. DSC-data analyzed with MicroCal Origin Software (VP-DSC version) are representative results of two independent experiments with standard deviations less than 5%. For Zeta experiments data analysis was processed using the instrumental Malvern’s DTS software to obtain the mean Zeta-potential and size value calculated from the means of three measurements of three independent experimental repetitions.

## Results

The two peptides used within this work reveal differences in their selective antitumor activity (see Table 1), while R-DIM-P-LF11-322 specifically induces apoptosis in cancer cells, DIM-LF11-318 exerts rapid killing of both, cancer and non-cancer cells [30]. The studies by Riedl et al. [30] also revealed correlation of specific antitumor activity with secondary structure and different permeabilization of membranes. To unveil further potential characteristics correlated with differences in selectivity extended model systems and *in vitro* studies were performed.

**Table 1:** Overview of peptide sequences and LC_50_ values of R-DIM-P-LF11-322 and DIM-LF11-318 [30].

Peptide	Sequence	LC_50_ (PI) [μM]	
		A375	NHDF
**R-DIM-P-LF11-322**	PFWRIRIRR-P-RRIRIRWFP-NH_2_	9.5 ± 0.3	65.1 ± 2.5
**DIM-LF11-318**	FWQRRIRRWRR-FWQRRIRRWRR-NH_2_	3.8 ± 0.5	2.2 ± 0.9

Data of toxicity studies determined by propidium iodide (PI)-uptake from at least seven experiments are presented as mean ± SD; A375, melanoma cell line; NHDF normal human dermal fibroblasts.

### Impact of phosphatidylserine and phosphatidylcholine on peptide activity and interaction

Due to the fact that cancer cells specifically expose the negatively charged lipid phosphatidylserine (PS) in the outer leaflet of the plasma membrane [7,15], the impact of the negatively charged PS as a target for the cationic peptides, the cancer-specific R-DIM-P-LF11-322 and the non-specific DIM-LF11-318 was studied. Model systems with liposomes composed of either single PS or mixtures containing PS and PC were used as cancer mimics, whereas liposomes composed of single PC were used as non-cancer “healthy” mimics. Cancer cells depleted of outer leaflet PS by the enzyme PS-decarboxylase (PSD) were used to confirm data gained from the model systems.

#### Changes of thermodynamic phase behavior of cancer cell and non-cancer cell mimicking liposomes

**Fig 1:**
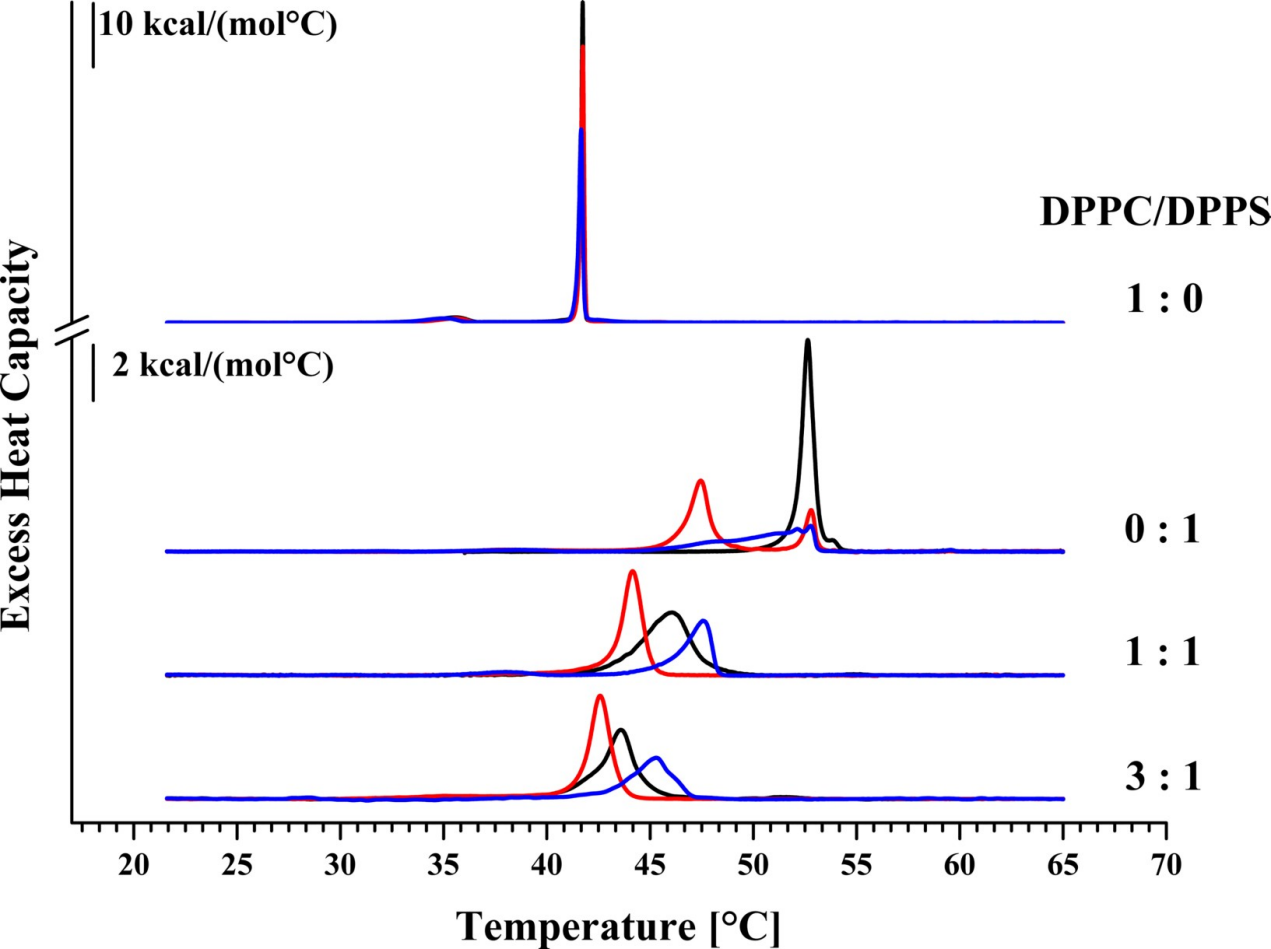
DSC thermograms of liposomes composed of DPPC and/or DPPS in absence and presence of R-DIM-P-LF11-322 or DIM-LF11-318. Liposomes of pure DPPC (1:0), pure DPPS (0:1) or DPPC/DPPS 1:1 and 3:1 (molar ratios; preparation freeze and thaw) were used to determine the effect of the two LF11 derivatives on the respective lipid systems (lipid-to-peptide molar ratio 25:1). Black represents thermograms in absence of peptide; red in presence of R-DIM-P-LF11-322 and blue in presence of DIM-LF11-318. For clarity, the DSC curves were displayed on the ordinate by an arbitrary increment. For analyzed data see Table 2.

To study the interaction of the peptides with the membrane lipids PS and/or PC, changes in the thermodynamic phase behavior of respective liposomes mimicking plasma membranes of cancer (DPPS, DPPC/DPPS 1:1 and 3:1, molar ratios) and non-cancer cells (DPPC) in presence of R-DIM-P-LF11-322 and DIM-LF11-318 (lipid-to-peptide molar ratio 25:1) were analyzed by Differential Scanning Calorimetry (DSC). Changes of the calorimetric enthalpy ΔH_cal_, the phase transition temperature T_m_ and the half width of the phase transition (T_1/2_) indicate potential perturbation of the respective mimics by the peptides (see Fig 1 and Table 2). As reported thermograms of DPPC and DPPS show two, respectively one characteristic phase transition(s) [25,28]. Consistent with recent reports [30] the thermotropic phase behavior of DPPC was not affected in the presence of R-DIM-P-LF11-322 indicating no interaction with the healthy mimic. Surprisingly the peptide DIM-LF11-318 which shows high activity on non-cancer cells *in vitro* only caused moderate membrane perturbation of DPPC liposomes indicated by a slight decrease of the ΔH_cal_ (Table 2). Contrary to R-DIM-P-LF11-322, DIM-LF11-318 though seems to be capable of entering PC liposomes without causing severe membrane damage, which has been shown in previous work by Riedl et al. [30]. However, severe membrane perturbation of the cancer mimic DPPS was observed in presence of both peptides. Upon incubation with R-DIM-P-LF11-322, the transition split into two peaks, presumably peptide enriched (lower T_m_) and peptide poor DPPS domains. A 25% decreased ΔH_cal_ and peptide affected lipid domains comprising 80% of the liposome with a phase transition temperature strongly decreased by 5.1°C and a highly increased half width confirm previous reports by Riedl et al. [30] revealing a strong perturbation of the cancer mimic DPPS by R-DIM-P-LF11-322. An even stronger effect on DPPS was seen in the presence of DIM-LF11-318 with a severe broadening (T_1/2_ 0.64°C to 3.24°C) of the transition and a decrease in ΔH_cal_ by almost 50%.

**Table 2:** Thermodynamic parameters of DPPC, DPPS and DPPC/DPPS 1:1 and 3:1 (molar ratios; freeze and thaw) liposomes in absence and presence of R-DIM-P-LF11-322 or DIM-LF11-318. For thermograms see Fig 1. DSC-data analyzed with MicroCal Origin Software (VP-DSC version) are representative results of two independent experiments with standard deviations less than 5%.

	ΔH_pre_ [kcal/mol]	T_pre_ [°C]	ΔH_m_ [kcal/mol]	T_m_ [°C]	T_1/2_ [°C]
**DPPC**	1.5	35.6	9.5	41.7	0.12
+ R-DIM-P-LF11-322	1.3	35.6	9.6	41.7	0.17
+ DIM-LF11-318	1.5	35.1	8.7	41.7	0.21
**DPPS**			9.9	52.6	0.64
+ R-DIM-P-LF11-322			7.5 (5.9/1.6)	47.5/52.8	1.07/0.54
+ DIM-LF11-318			5.3	52.8	3.24
**DPPC/DPPS (1:1)**			10.6	46.1	2.59
+ R-DIM-P-LF11-322			8.2	44.2	1.07
+ DIM-LF11-318			5.2	47.6	1.43
**DPPC/DPPS (3:1)**			9.5	43.6	1.51
+ R-DIM-P-LF11-322			8.8	42.6	1.05
+ DIM-LF11-318			6.2	45.4	2.23

Additionally, cancer cell mimicking liposomes composed of DPPC/DPPS 1:1 and 3:1 molar ratios were studied (Fig 1). The 1:1 mixture showed one broad main phase transition at 46.1°C. Addition of R-DIM-P-LF11-322 and DIM-LF11-318 resulted in a decrease of the main transition enthalpy though to different extent. In presence of R-DIM-P-LF11-322, the enthalpy was reduced by 20%. Furthermore, a severe temperature shift to lower temperature by 1.9°C occurred, indicating that the peptide preferentially perturbs the higher chain melting DPPS proportion of the mixture leaving DPPC enriched liposomes in the gel phase unaffected. This finding is also confirmed by an increase in cooperativity by more than 50%. Addition of DIM-LF11-318 resulted in an even stronger reduction of the enthalpy by 50% (from 10.6 kcal/mol to 5.2 kcal/mol). However contrary to R-DIM-P-LF11-322, DIM-LF11-318 induced a shift of the phase transition to higher temperatures (by 1.5°C), which again indicates a strong perturbation of the lipid mixture, though in this case with also a presumable perturbation of a proportion of DPPC. With decreasing PS ratios (DPPC/DPPS 3:1) the effect of R-DIM-P-LF11-322 decreases, nevertheless, a by 1°C lower T_m_ still indicates a strong interaction of the peptide with the now reduced proportion of 25 mol% DPPS. DIM-LF11-318 again induces a stronger effect leading to a reduction of the phase transition enthalpy by 35%, indicating perturbation of both lipids, DPPS (25 mol%) and DPPC (75 mol %). Again, the increase of the main transition temperature by 1.8°C (to 45.4°C) indicates the possible perturbation of DPPS and DPPC liposomes by the peptide. Addition of R-DIM-P-LF11-322 leads to a more homogenous phase transition at decreased temperatures (T_1/2_ = 1.51°C in absence and 1.05°C in presence of R-DIM-P-LF11-322), confirming specific interaction with DPPS. In contrast, addition of DIM-LF11-318 results in an increase of T_1/2_ by 0.7°C, indicating an interaction with both components of the mixture by the non-specific peptide.

#### Electrostatic and hydrophobic interactions of peptides with cancer cell mimicking liposomes

**Fig 2:**
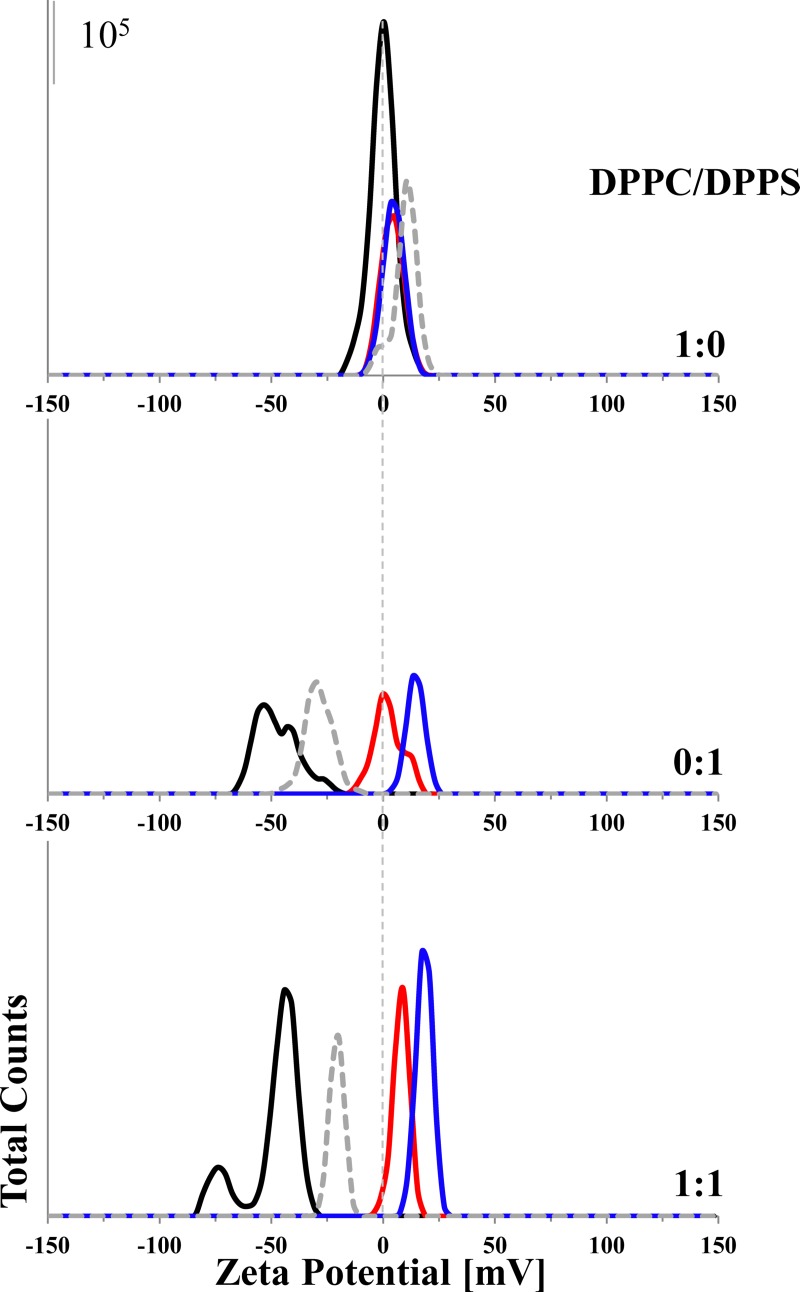
Zeta potential of different liposomes in presence and absence of R-DIM-P-LF11-322, DIM-LF11-318 or CaCl_2_. The zeta potentials of pure DPPC and DPPS liposomes as well as of DPPC/DPPS 1:1 (molar ratio) liposomes were determined in absence (black) and presence of R-DIM-P-LF11-322 (red), DIM-LF11-318 (blue) (lipid-to-peptide molar ratio 25:1) or CaCl_2_ [1 mM] (dashed gray). While there is no significant change in zeta potential of DPPC liposomes in presence of peptides/calcium, addition reduces the pre-existing negative zeta potential of liposomes when DPPS is present. The data indicate an interaction of the peptides/calcium with the negatively charged lipid. Representative Zeta-distribution is shown of three independent experiments. For analyzed data see S1 Table. Means were calculated of data of 30 measurements in two experimental repetitions.

Zeta potential and size measurements were performed with DPPC, DPPS and DPPC/DPPS (1:1; molar ratio) liposomes in presence and absence of R-DIM-P-LF11-322, DIM-LF11-318 (lipid-to-peptide molar ratio 25:1) or CaCl_2_ [1 mM], respectively (see Fig 2, S1 Table) in order to study the impact of lipids on peptide-membrane interactions. For these experiments, peptides were added right before measurements to extruded liposomes in order to analyze the first steps of interaction of the peptides with plasma membranes of cancer and non-cancer cells.

While DPPC liposomes, mimicking healthy cells, exhibited a neutral zeta potential (~0 mV), cancer cell mimicking DPPS liposomes as well as 1:1 mixtures of both lipids showed a negative zeta potential of about -50 mV. These data are in accordance with zeta potentials shown for non-cancer cells and cancer cells, respectively [30]. Addition of R-DIM-P-LF11-322, DIM-LF11-318 or CaCl_2_ did not lead to any significant changes of the zeta potential of the DPPC liposomes, indicating absence of any (electrostatic or hydrophobic) interaction. On the contrary, addition of R-DIM-P-LF11-322 to DPPS liposomes strongly shifted the zeta potential from—60 mV to ~0 mV, so the specific peptide was able to completely neutralize the negatively charged surface. This indicates electrostatic interaction between the positive charges of the peptide and the negative charges of the liposomes. Addition of DIM-LF11-318 led to an even stronger shift of the zeta potential to even positive values (+18 mV) supposing beyond electrostatic probably also hydrophobic interactions. Similar effects were observed when R-DIM-P-LF11-322, DIM-LF11-318 or CaCl_2_ were added to DPPC/DPPS 1:1 liposomes. The presence of R-DIM-P-LF11-322 induced neutralization of the negative surface potential. The presence of DIM-LF11-318 induced more positive zeta potentials up to + 20 mV, indicating electrostatic and hydrophobic interactions with both lipids, DPPS and DPPC (see suppl., S1 Table).

Regarding the size of the liposomes there was no significant change observed for DPPC when peptides were added (see suppl., S1 Table). In contrast, addition of R-DIM-P-LF11-322 or DIM-LF11-318 to DPPS or to DPPC/DPPS (1:1) liposomes did not only lead to changes in the zeta potential but also to a significant increase of size. This indicates possible aggregation/fusion of the liposomes as a consequence of peptide interaction and neutralization of the surface. Interestingly DIM-LF11-318 even induced two different size populations. Addition of Ca^+2^ at 1 mM had no effect on size. Therefore, interaction of molecules with liposomes shown by change of the zeta potential does not naturally lead to an increase in size. However, the amount of Ca^2+^ was shown to be not sufficient to completely neutralize the negative surface charge of the DPPS liposomes, which might probably affect aggregation. In general, a correlation between the changes of the zeta potential due to interactions of peptides with membranes and an increase of the liposomal size could be observed for both peptides.

### Effect of PS-exposure on peptide activity and interaction *in vitro*–Depletion of PS exposed by cancer cells by PS-decarboxylase PkPSD

**Fig 3:**
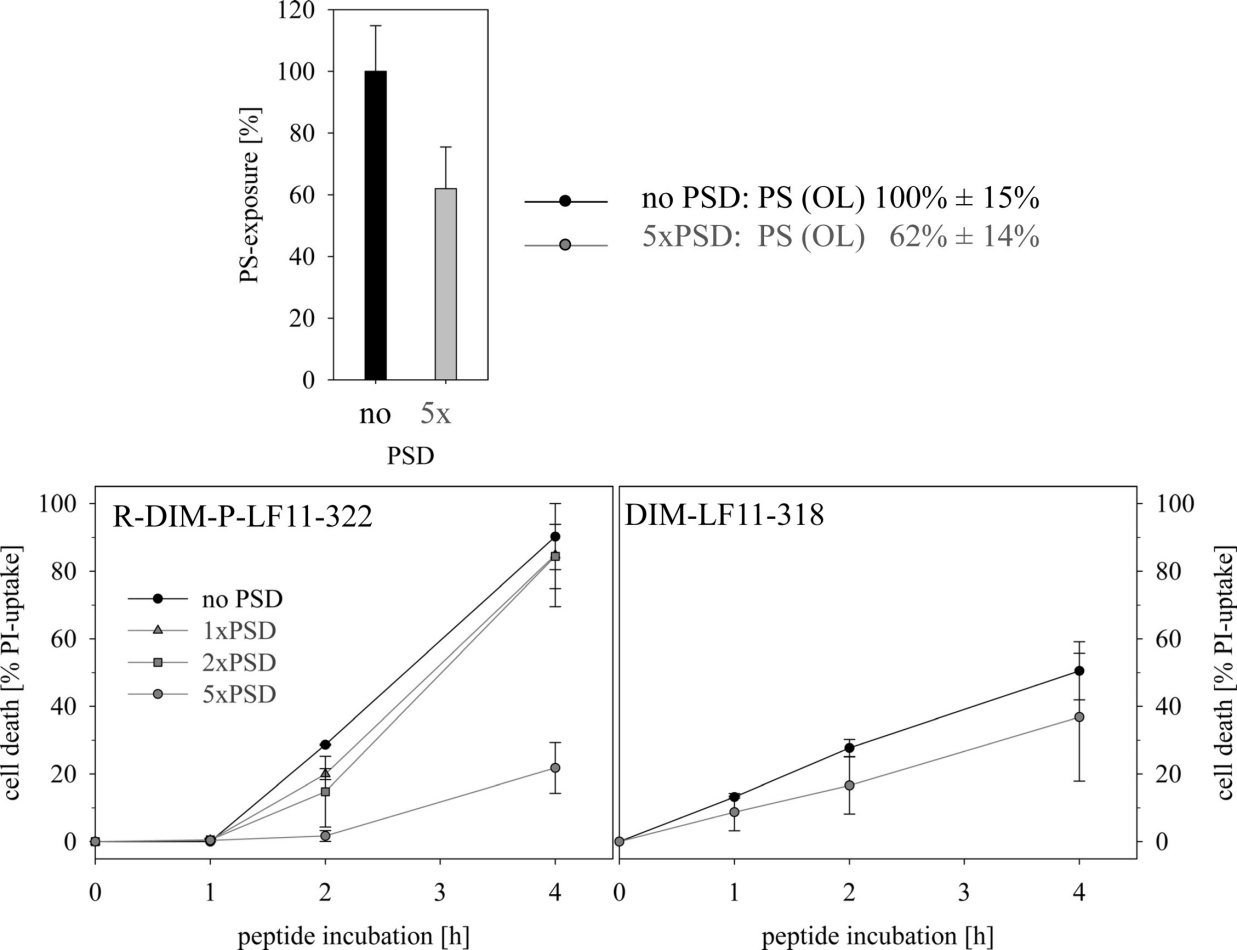
**Effect of PS-exposure on cell death induced by 5 μM of peptides R-DIM-P-LF11-322 (left) or DIM-LF11-318 (right) in the presence of normal (100%) levels of PS exposed by melanoma cell line A375 (no PSD) or in the presence of reduced levels of PS (1xPSD, 2xPSD and 5xPSD (1xPSD 2,7 μg/10^5^ cells) (at least 3 experiments). 5xPSD (13,4 μg/10^5^ cells) leads to an approximate reduction of PS-exposure in the outer leaflet (OL) of the plasma membrane of A375 by 40% (at least 2 experiments)**. R-DIM-P-LF11-322 in contrast to DIM-LF11-318 shows a significant reduction in killing of melanoma cells in the presence of reduced PS-exposure. PS-exposures was determined by binding to Annexin V-Alexa Fluor 488 (λ_ex_ = 488 nm and λ_em_ = 530 nm). For the measurements serum free media was used to prevent proteolysis of PSD during 30 min of incubation. PSD, PS-decarboxylase PkPSD.

A further experiment to support the importance of PS as a target for the antitumor peptides was to reduce the level of PS exposed on the surface of the cancer cells by the enzyme PS-decarboxylase (PkPSD), which can convert PS to PE (phosphatidylethanolamine) and observe potential effects on peptide activity. PkPSD cannot enter the cell due to its size and therefore only reduces surface PS. Indeed, as can be seen in Fig 3 within 30 min upon treatment with 5xPkPSD (13.4 μg) a significant reduction of the original PS-level exposed by the melanoma cells by 40% was reached. To minimize supply with PS from the inner to the outer leaflet by flip flop, experimental observation of changes in peptide activity was further restricted to four hours. Interestingly the reduction of the surface-exposed PS-levels showed different effects on the two peptides studied. Whereas the reduced PS exposed on melanoma cells dramatically reduced activity of the cancer specific R-DIM-P-LF11-322 (by 75%), the non-specific DIM-LF11-318 was not significantly affected (Fig 3**)**. These differences were distinct at low peptide concentrations (5 μM) and in the absence of serum, which should prevent proteolytic degradation of PSD. Interestingly, R-DIM-P-LF11-322 showed increased activity in the absence of serum. A possible explanation is that the peptide is prone to proteolytic degradation. In summary the experiment revealed a strong need for “cancer” PS exposed for effective killing by the cancer specific R-DIM-P-LF11-322 and no limiting factor for the killing efficacy of DIM-LF11-318, which can probably also target other components of the cell membrane. This confirms and expands our model proposed previously for the different action of the two peptides with cancer and non-cancer membranes [30].

### Impact of Cholesterol on peptide activity and interaction

#### Cholesterol distribution in cancer and non-cancer membranes

**Fig 4:**
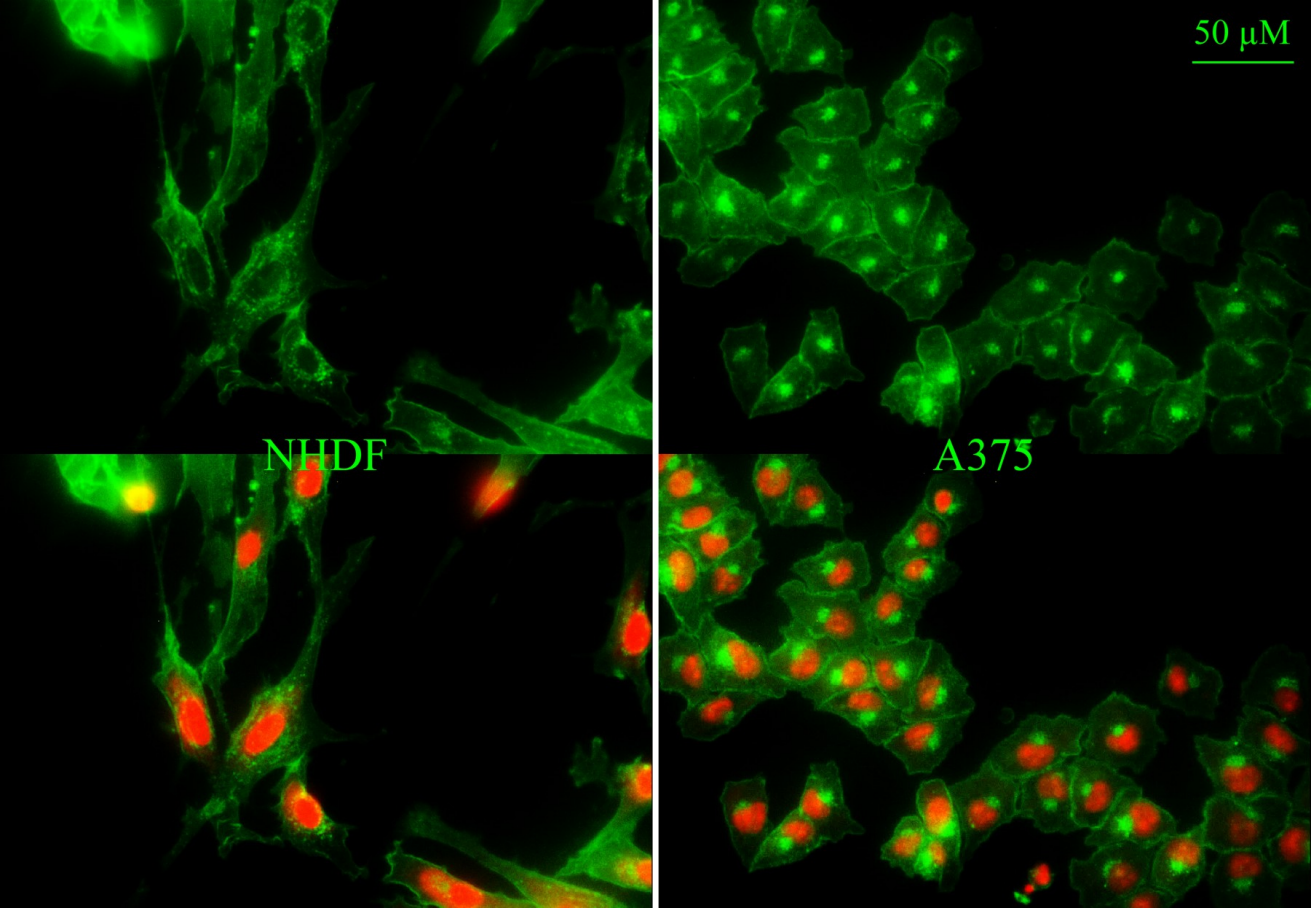
**Distribution of cholesterol in membranes of normal human dermal fibroblasts NHDF (left) and melanoma cells A375 (right).** Cholesterol was stained with filipin (green) and nucleus with PI (red). Overlay at the bottom. Pictures are representative results of more than three independent experiments.

To study potential influence of further components of the plasma membrane, the model systems were now extended by the neutral lipid cholesterol. Cholesterol is an important eukaryotic membrane compound controlling e.g. permeability and stability of the membranes [36]. The plasma membrane comprises the highest amount of cholesterol followed by endosomes, the Golgi apparatus and mitochondria [48]. Indeed, staining of cholesterol (green; filipin) of normal human dermal fibroblasts NHDF (Fig 4, left) revealed cholesterol localized to the plasma membrane and compartments comprised of fine dots surrounding the nucleus (red; PI) resembling mitochondria and/or endosomes. The melanoma cells A375 (Fig 4, right) also showed a distinct staining of plasma membrane cholesterol. The strong intracellular cholesterol localization however was different to that of NHDF, mainly one large spot found at one side of the nucleus was seen, an appearance typical for the Golgi apparatus (see also paragraph “effect of peptides on morphology of cancer cell membranes of living cells”, staining of Golgi apparatus) [49].

Cholesterol has been discussed to exercise influence on the membrane interaction of peptides. Matsuzaki et al. [50] reported that the presence of cholesterol reduced the effects of magainin on membranes. Thus in the following a possible difference in the effect of cholesterol on the cancer specific R-DIM-P-LF11-322 and the non-specific DIM-LF11-318 was studied.

#### Different impact of cholesterol on peptide activity and interaction

**Fig 5:**
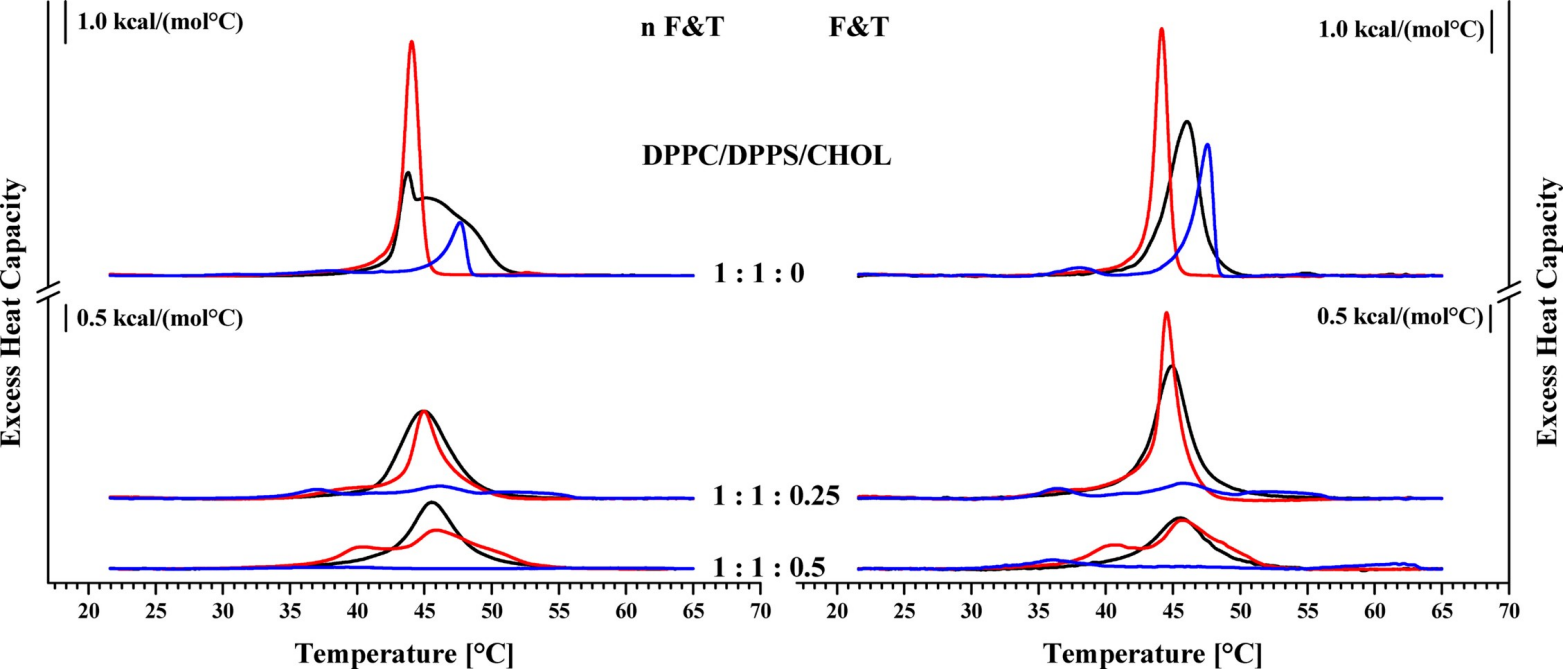
**DSC thermograms in absence and presence of R-DIM-P-LF11-322 or DIM-LF11-318 of non-freeze and thaw (n F&T, domains; left) and freeze and thaw (F&T, no (less) domains; right) preparations of DPPC/DPPS liposomes with increasing amounts of cholesterol (1:1:0; 0.25; 0.5 DPPC/DPPS/Chol; molar ratios)**. Black represents measurements without peptide; red corresponds to measurements in presence of R-DIM-P-LF11-322 and blue in presence of DIM-LF11-318. For clarity, the DSC curves were displayed on the ordinate by an arbitrary increment. For analyzed data see Table 3.

To elevate potential changes in the interaction of peptides with membranes containing cholesterol, cancer mimics composed of DPPC and DPPS without (1:1:0 molar ratio) and stepwise increasing amounts of cholesterol (1:1:0.25, 1:1:0.5; molar ratios) were studied in absence and presence of peptides R-DIM-P-LF11-322 or DIM-LF11-318 (lipid-to-peptide molar ratio 25:1), respectively. The liposomes with and without cholesterol were prepared by a freeze and thaw preparation (F&T) to yield homogeneous distribution of different lipid species (Fig 5 right). In addition, a second set of experiments included the preparation without freeze and thaw (nF&T) to yield an inhomogeneous, more “natural” lipid distribution, to study a possible impact of *in vivo* occurring lipid domains on peptide activity (Fig 5 left). DSC analyses of liposomes prepared by the two different methods confirmed the existence of additional domains in case of the nF&T method (macroscopic domains). The thereby prepared liposomes were to different extent enriched in PS or PC, resulting in a splitting of the transition (1:1:0). The F&T preparation yielded liposomes exhibiting a more cooperative transition due to a more homogeneous distribution of PS and PC. With increasing levels of cholesterol the enthalpy of phase transition gets reduced which is conform with literature reporting that cholesterol increases the fluidity of lipids in the gel phase [51]. Increasing amounts of cholesterol also reduced formation of macroscopic domains in the gel phase. As described before (Fig 1) addition of both peptides to liposomes composed of PC and PS (absence of cholesterol) resulted in a different perturbation of the components of the mixture with a main interaction of R-DIM-P-LF11-322 with the cancer mimic DPPS (decrease of T_m_) and a perturbation by DIM-LF11-318 with both lipids of the mixture DPPC and DPPS (increase of T_m_) (Fig 5; Table 3). Interestingly the existence of macroscopic domains had no impact on the effect of R-DIM-P-LF11-322 (strong interaction with PS, ΔH_m; domains and no domains_ -23%), whereas DIM-LF11-318 even showed an increased perturbation of the PS and PC lipids in the presence of large domains (ΔH_m;domains_ -80%, ΔH_m;no domains_ -51%). The presence of cholesterol now induced a decrease of the effect of R-DIM-P-LF11-322, indicated by a smaller reduction of the enthalpy and temperature of the phase transition. However, the presence of domains seemed to support the activity of the peptide (no domains vs domains: -23% vs -23% (1:1:0); -14% vs- 23% (1:1:0.25); +19% vs -8% (1:1:0.5)) contrary to the thermotropic phase behavior in absence of cholesterol (1:1:0), where no difference in the activity was seen. In the presence of 11 and 25 mol% cholesterol (1:1:0.25 and 1:1:0.5) R-DIM-P-LF11-322 induced a phase separation into two populations with increasing proportions of peptide and PS poor (PC enriched) liposomes at lower temperatures (40°C) and decreasing remaining peptide poor and less affected lipid mixtures (46°C; 46.6°C). An enrichment of cholesterol in one of the lipid domains is possible, but cannot be confirmed by the data obtained with DSC.

**Table 3:** Thermodynamic parameters of non-freeze and thaw (first row) and freeze and thaw (second row) preparations of liposomes of DPPC/DPPS/Cholesterol 1:1:0, 1:1:0.25 and 1:1:0.5 (molar ratios) in absence and presence of R-DIM-P-LF11-322 or DIM-LF11-318. Deconvolution was used for analyses of data of overlapping phase transitions. For thermograms see Fig 5. DSC-data analyzed with MicroCal Origin Software (VP-DSC version) are representative results of two independent experiments with standard deviations less than 5%.

	ΔH_m_ [kcal/mol]	T_m_ [°C]	T_1/2_ [°C]
**DPPC/DPPS/Cholesterol (1:1:0)**			
*non freeze and thaw (domains)*	*11*.*4 (0*.*6/10*.*8)*	*43*.*7/45*.*6*	*0*.*39/5*.*27*
freeze and thaw	10.6	46.1	2.59
+R-DIM-P-LF11-322			
*non freeze and thaw (domains)*	*8*.*7 (-23%)*	*44*.*0*	*1*.*20*
freeze and thaw	8.2 (-23%)	44.2	1.07
+ DIM-LF11-318			
*non freeze and thaw (domains)*	*2*.*3 (-80%)*	*47*.*7*	*1*.*46*
freeze and thaw	5.2 (-51%)	47.6	1.43
**DPPC/DPPS/Cholesterol (1:1:0.25)**			
*non freeze and thaw (domains)*	*6*.*2*	*44*.*9*	*4*.*20*
freeze and thaw	7.7	44.8	2.87
+R-DIM-P-LF11-322			
*non freeze and thaw (domains)*	*4*.*8 (-23%)*	*44*.*9*	*2*.*28*
freeze and thaw	6.6 (-14%)	44.5	1.40
+ DIM-LF11-318			
*non freeze and thaw (domains)*	*1*.*8 (0*.*4/0*.*9/0*.*5) (-71%)*	*36*.*9/46*.*3/51*.*4*	*3*.*48/4*.*98/5*.*87*
freeze and thaw	2.0 (0.4/1.1/0.5) (-74%)	36.5/45.8/51.7	3.05/4.35/5.97
**DPPC/DPPS/Cholesterol (1:1:0.5)**			
*non freeze and thaw (domains)*	*5*.*3*	*45*.*6*	*3*.*95*
*freeze and thaw*	4.2	45.5	4.57
+R-DIM-P-LF11-322			
*non freeze and thaw (domains)*	*4*.*9 (1*.*6/3*.*3) (-8%)*	*40*.*1/46*.*6*	*5*.*09/5*.*31*
freeze and thaw	5 (1.3/3.7) (+19%)	40.2/46.0	3.82/4.85
+ DIM-LF11-318			
*non freeze and thaw (domains)*	*0*.*3 (0*.*2/0*.*1) (-94%)*	*39*.*3/56*.*9*	*8*.*12/3*.*42*
freeze and thaw	1.4 (0.7/0.3/0.4) (-67%)	36.1/45.3/61.5	4.73/4.59/5.43

Contrary to the reducing effect of cholesterol on the lipid perturbation by D-DIM-P-LF11-322, the strong activity of peptide DIM-LF11-318 was not affected by cholesterol. Since in the presence of cholesterol and DIM-LF11-318 nearly no phase transition was observable anymore, the effect of domains in presence of cholesterol on the peptide activity was hard to determine. However, it can be proposed that DIM-LF11-318 strongly perturbs membranes composed of PS and PC in both, the absence and presence of cholesterol and also domains. In absence of cholesterol, the increasing effect of domains is further striking.

#### Different impact of cholesterol on electrostatic and hydrophobic interactions of peptides with cancer membrane mimics

**Fig 6:**
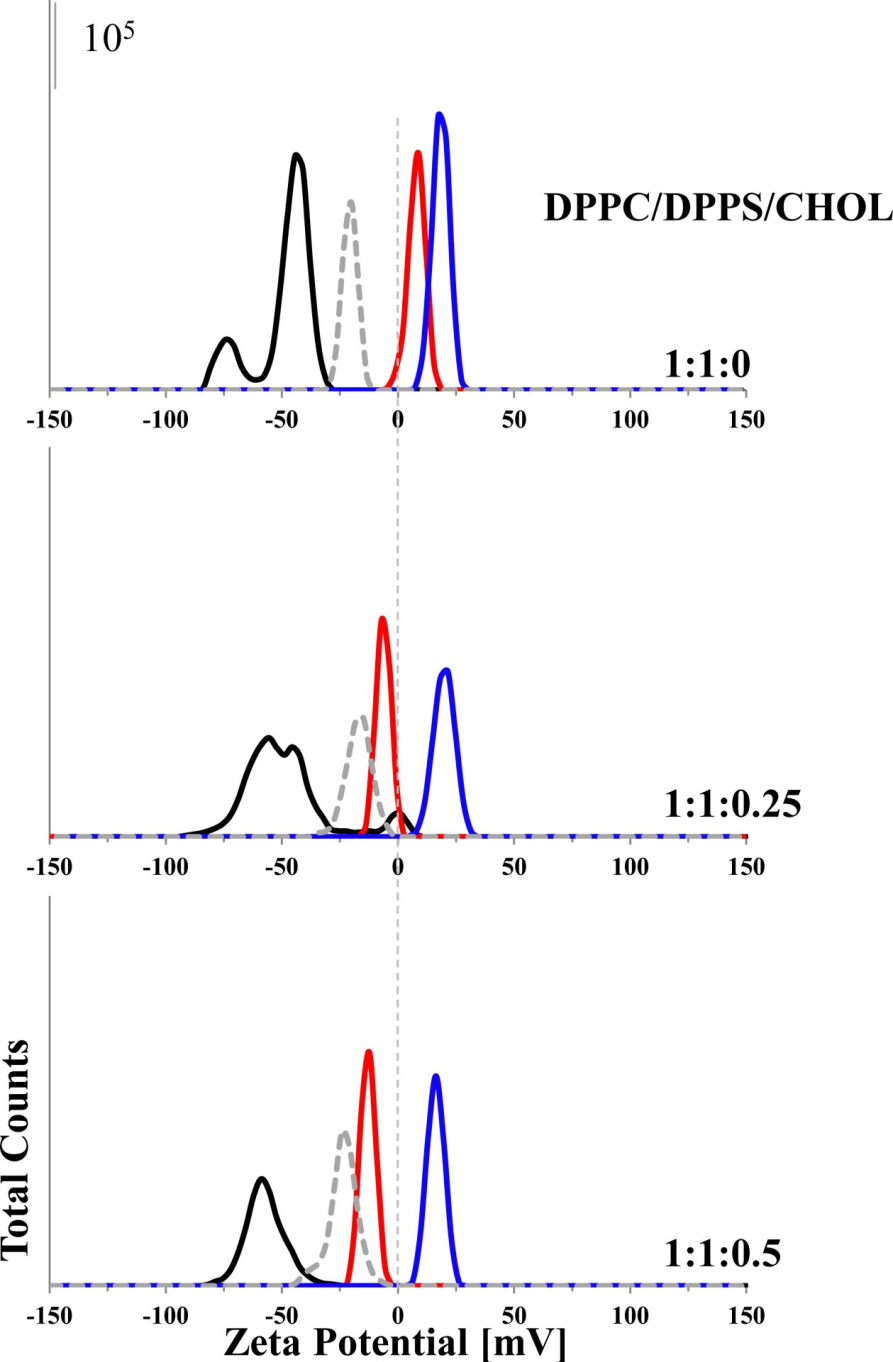
Zeta potential of different liposomes in presence and absence of R-DIM-P-LF11-322, DIM-LF11-318 or CaCl_2_. The zeta potentials of liposomes composed of DPPC/DPPS 1:1 (molar ratio) with increasing ratios of cholesterol (1:1:0; 1:1:0.25; 1:1:0.5) were determined in absence (black) and presence of R-DIM-P-LF11-322 (red) and DIM-LF11-318 (blue) (lipid-to-peptide molar ratio 25:1) or CaCl_2_ [1 mM] (dashed gray). Peptide or calcium addition reduced the negative zeta potential of the respective liposomes. This indicates an interaction of the peptides/calcium with the negatively charged liposome. In presence of cholesterol the effect of R-DIM-P-LF11-322 gets reduced, while DIM-LF11-318 remains unaffectedly strong in its activity. Figure shows a representative result of three independent experiments. For analyzed data (means of 30 runs of 3 measurements of three experiments), see S1 Table.

To determine a potential influence of cholesterol on peptide activity zeta potentials were analyzed in presence of increasing cholesterol amounts with liposomes composed of DPPC/DPPS/Cholesterol with molar ratios of 1:1:0, 1:1:0.25 and 1:1:0.5 in presence and absence of R-DIM-P-LF11-322, DIM-LF11-318 (lipid-to-peptide molar ratios 25:1) or CaCl_2_ [1 mM]. As can be seen in Fig 6 and S1 Table the zeta potentials of the 1:1:0, 1:1:0.25 and the 1:1:0.5 liposomes without peptide were in the range of -50 mV to -60 mV. In presence of R-DIM-P-LF11-322 the zeta potential of liposomes with increasing cholesterol shifted from +5 mV (1:1:0) to -3 mV (1:1:0.25) to -15 mV (1.1:0.5). This might indicate less electrostatic interaction of the cancer specific peptide with increasing ratios of cholesterol. Contrary, the zeta potentials of the different liposomes with increasing amounts of cholesterol in presence of DIM-LF11-318 remained mainly unaffected within the range of +20 mV (1:1:0 and 1.1:0.25) to +16 mV (1:1:0.5), again indicating that the membrane interaction of DIM-LF11-318 is not affected by cholesterol. As discussed before the first interaction of R-DIM-P-LF11-322 with the target membrane seems to be electrostatic and that of DIM-LF11-318 electrostatic and hydrophobic, due to the shift to positive values in presence of the latter. Addition of the peptides further led to a strong increase in size of the particles, indicating that there was aggregation/fusion induced by the peptides. Ca^+2^-addition did not lead to an increase in size. However, size measurements were only suitable for rough size approximations due to high polydispersity (size) of samples. Determination of accurate size differences between liposomes with different lipid compositions was therefore not possible. However, it was possible to deduce changes upon peptide addition. In general, a correlation of changes in the zeta potential and increase of size could be seen (S1 Table).

#### Impact of cholesterol on peptide induced lipid distribution on GUVs

**Fig 7:**
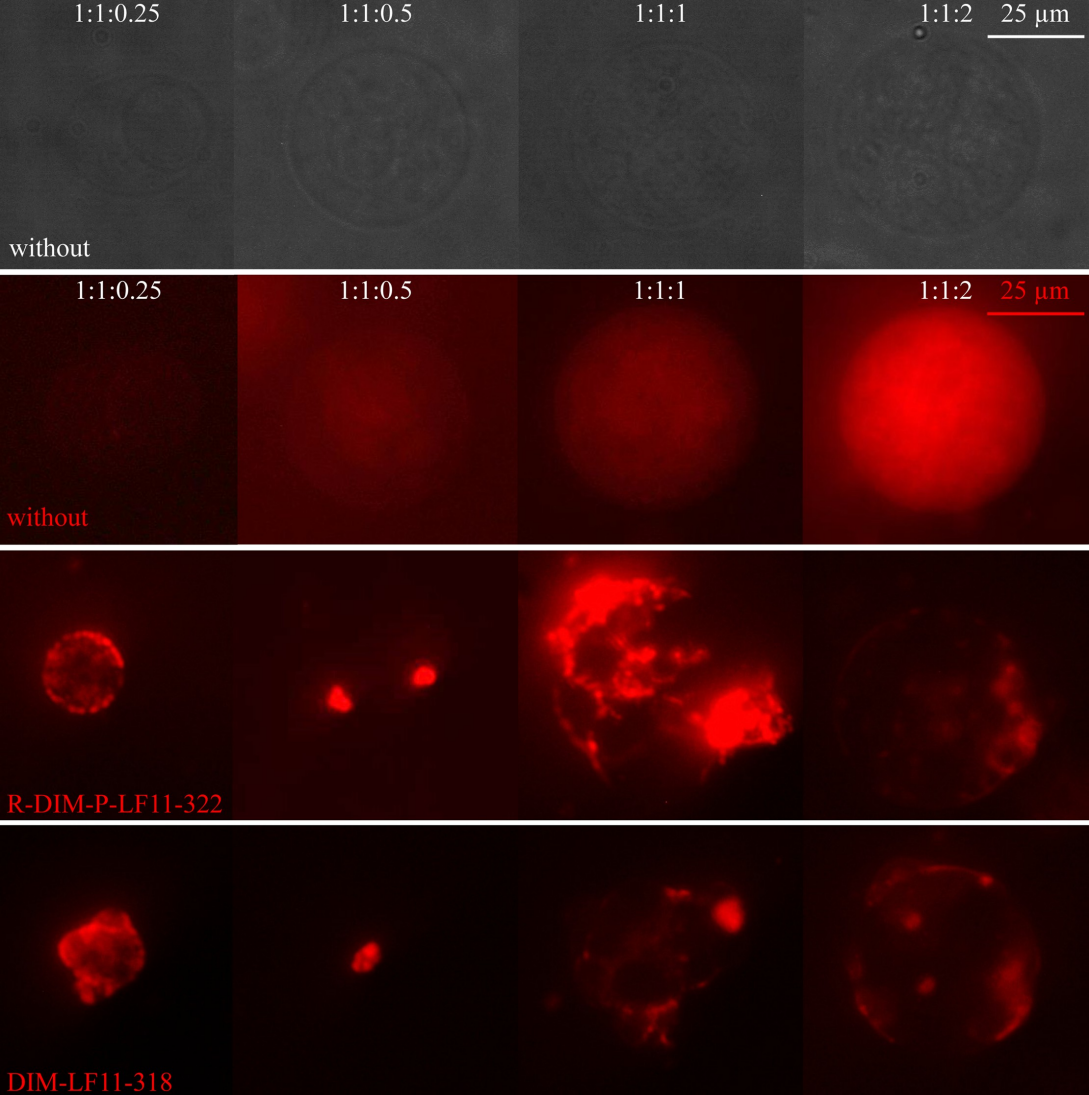
Giant unilamellar vesicles of DPPC/POPS/Cholesterol with increasing amounts of cholesterol in absence and presence of R-DIM-P-LF11-322 and DIM-LF11-318. GUVs consisting of DPPC/POPS/Cholesterol (1:1:0.25, 1:1:0.5, 1:1:1 and 1:1:2 molar ratios) + Rhodamine-DOPE (red) were generated using electro-formation. Visualization followed in absence of peptides (1^st^ (bright field) and 2^nd^ row), in presence of R-DIM-P-LF11-322 (3^rd^ row) or DIM-LF11-318 (4^th^ row) (50 μM). Peptide incubation for 1:1:1 and 1:1:2 was 30 minutes, 1:1:0.25 and 1:1:0.5 were visualized immediately due to strong destruction. Lipid-to-peptide molar ratios were 3:1. Bright areas show domains of fluid lipids. Brightness and contrast were equally enhanced for GUVs in presence of peptides. Brightness of pictures in absence of peptide (1^st^ row) had to be enhanced twice (1:1:1) or 1.5-fold (1:1:0.5 and 1:1:1) due to the weak signal. Pictures were taken of a multiple set of experiments (at least three independent preparations) and are representative for the respective experimental conditions.

To study potential changes upon peptide addition in distribution or (dis)-appearance of domains on liposomal models with various cholesterol content, GUVs were generated as described in the Methods section. Fig 7 shows the impact of the respective ratios of cholesterol of GUVs on interaction with the two peptides R-DIM-P-LF11-322 or DIM-LF11-318 (DPPC/POPS/Cholesterol 1:1:0.25, 1:1:0.5, 1:1:1 and 1:1:2; molar ratios).

In absence of peptides, fluorescence was weak, but increased with the amount of cholesterol. The distribution of domains was homogeneous. With increasing amounts of cholesterol addition of the two peptides had comparable effects, a strong induction of fluid domains (all mixtures), destruction of liposomes or decrease in size (1:1:0.25 and 1:1:0.5), fusion (1:1:1), and reduced though still dramatic effects on the lipid and domain distribution at highest cholesterol levels (1:1:2). GUVs of 1:1:0.25 and 1:1:0.5 needed to be analyzed immediately after peptide addition, due to the strong and fast destruction of these mixtures by the peptides.

Increasing amounts of cholesterol led to an increase in size of the GUVs. Presence of peptides however reduced the size of GUVs at lower cholesterol levels (1:1:0.25 and 1:1:5).

1:1:0.25 GUVs partially appeared to be oval shaped (Fig 7, bright field).

#### Cholesterol depletion and its effect on peptide activity

**Fig 8:**
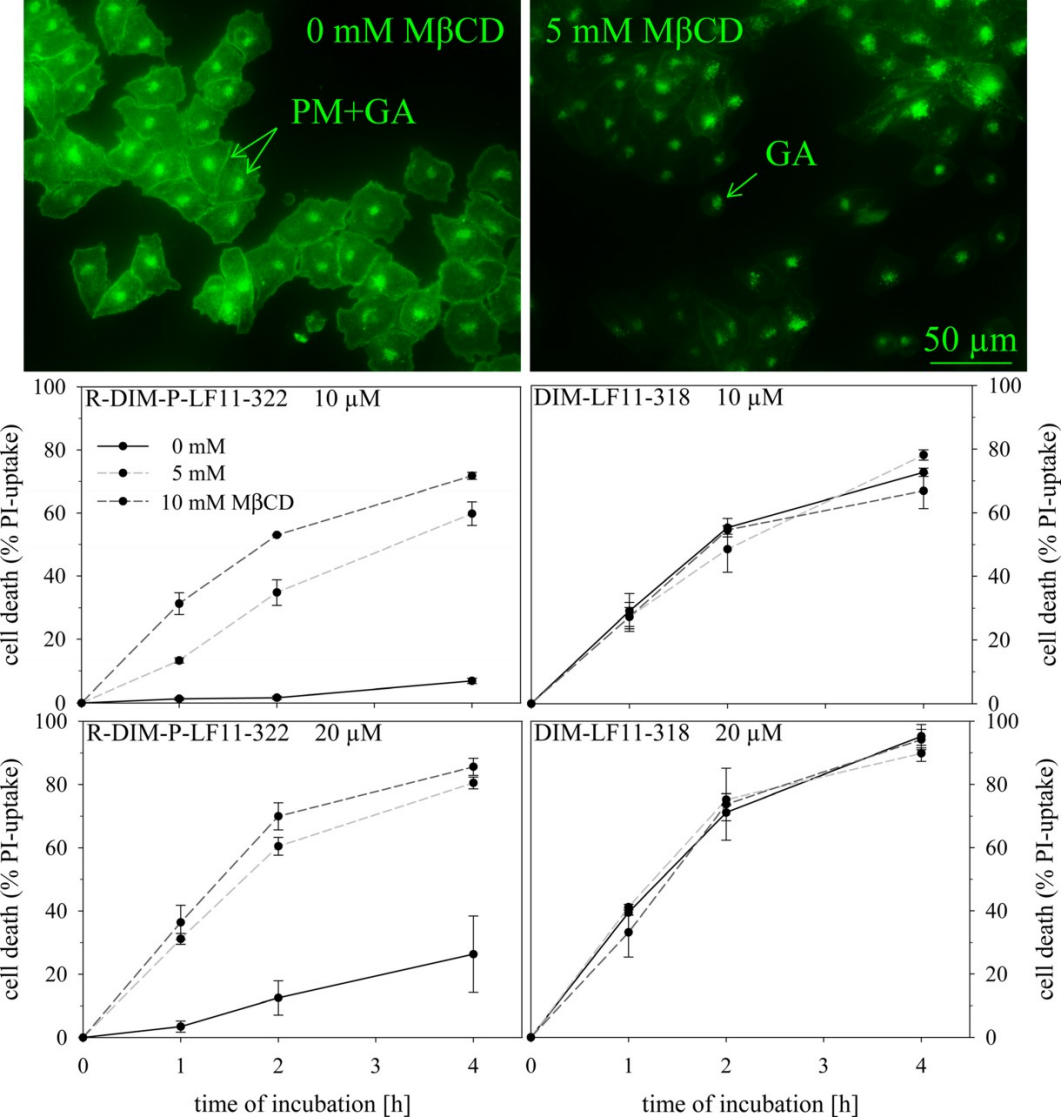
Influence of cholesterol depletion of plasma membrane (PM) on killing efficiency of peptides. A375 cells were treated with 0 mM, 5 mM and 10 mM of cholesterol depletion agent MβCD before peptide incubation. Top: Cholesterol of plasma membrane PM (left) was already efficiently depleted in the presence of 5 mM MβCD (right) as seen by microscopic inspection upon staining with filipin (green). Only intracellular cholesterol remained, presumably located to the Golgi apparatus (GA). Bottom: Upon peptide incubation (10 μM and 20 μM DIM-LF11-322, left; 10 and 20 μM R-DIM-P-LF11-318, right) the PI-uptake of cancer cells, which is proportional to the percentage of killed cells, was measured over four hours. Black represents cell death of A375 in presence of peptides without MβCD treatment, where plasma membrane cholesterol is present. Light gray and gray dashed lines represent cells treated with 5 mM and 10 mM MβCD, respectively. The result indicates a reduction of R-DIM-P-LF11-322 efficacy in presence of PM cholesterol, whereas toxicity of DIM-LF11-318 seems independent on PM cholesterol. Both peptides do not enter cells by clathrin or caveolae mediated endocytosis. Pictures are representative results of three independent experiments. PI-uptake is shown as a mean ± SEM of three independent experiments.

Cholesterol has been discussed to exert a protective role in membranes against damage caused by host defense antitumor peptides [50]. To study potential impact of plasma membrane cholesterol on activity of the two peptides towards malignant melanoma cells, they were treated with different amounts of the cholesterol depletion agent methyl-beta-cyclodextrin (MβCD; 0 mM, 5mM and 10 mM) before peptide addition. Filipin staining of untreated A375 cells revealed cholesterol distribution in the plasma membrane (PM) and intracellular organelles (presumably the Golgi apparatus GA) (Fig 8, left). Already in presence of 5 mM MβCD cholesterol was efficiently depleted from the PM, only intracellular cholesterol (GA) remained (Fig 8, right). A potential cell death induced by MβCD or absence of PM cholesterol could be excluded due to absence of PI-uptake. Furthermore, it was proven that even after two hours no significant amounts of intracellular cholesterol were re-transported to the PM or taken up from the surrounding media (suppl., S1 Fig). Cytotoxicity was studied in presence (0 mM MβCD) and absence (5 and 10 mM MβCD) of cholesterol with R-DIM-P-LF11-322 or DIM-LF11-318 (Fig 8). Depletion of cholesterol showed dramatic effects on the specific peptide R-DIM-P-LF11-322. Activity was significantly increased against cholesterol depleted cells by 5-10-fold (10 and 20 μM peptide). E.g., upon four hours of peptide incubation (10 μM) an increase of killing efficiency by R-DIM-P-LF11-322 from 7% to 70% in cholesterol depleted cells was observed. In contrast, DIM-LF11-318 was not affected in its activity by cholesterol, exhibiting strong toxicity on melanoma cells in presence and absence of PM cholesterol. Therefore, a potential protective role of cholesterol seems only being exerted against cancer specific peptides as R-DIM-P-LF11-322. However, in cancer cells PS exposure is the driving force for potent killing (especially after longer time periods) even in presence of cholesterol.

Since MβCD also acts as inhibitor of clathrin and caveolae mediated endocytosis [52,53], the thereby mediated uptake can be excluded for both peptides.

### Effect of peptides on morphology of cancer cell membranes of living cells

**Fig 9:**
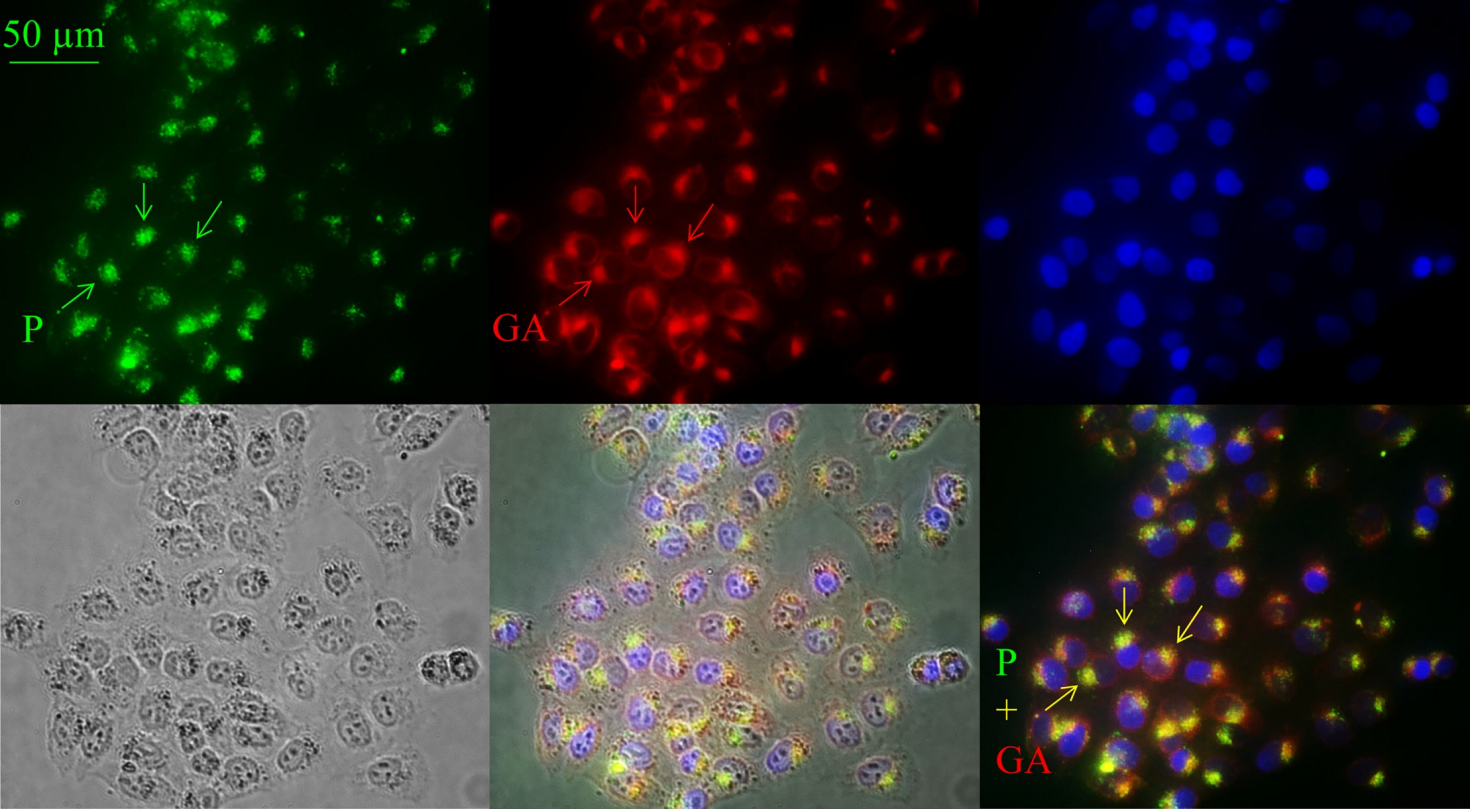
Localization of (5–6)-FAM-R-DIM-P-LF11-322 in A375. Melanoma cells (A375) were incubated for two hours with 10 μM fluorescently labeled ((5–6)-FAM-) R-DIM-P-LF11-322. Peptide localization is shown in green, Golgi staining by BODIPY ceramide in red and nuclear staining by NucBlue (blue). Bright field and overlays are illustrated at the bottom. Peptide and Golgi show overlapping staining (yellow) close to the nucleus, suggesting intracellular localization of the peptide to the Golgi apparatus. Peptide (P); Golgi apparatus (GA). Pictures are representative results of six independent experiments.

As shown by Riedl et al. [30] the cancer specific peptide R-DIM-P-LF11-322 triggers apoptosis upon four to eight hours, whereas the non-specific DIM-LF11-318 induces necrosis and fast lysis of cancer cells. Apoptosis demands entrance of peptide into cells and interaction with intracellular targets as mitochondria or Golgi [54,55], necrosis mainly requires interaction with the plasma membrane. To visualize a potential (intra)cellular interaction site of the peptide R-DIM-P-LF11-322 the (5–6)-FAM (green) labeled peptide was studied for its localization in A375 melanoma cells. It was observed that within two hours of incubation the peptide (green, arrows) enters the cell and localizes in an area next to the nucleus (blue) (Fig 9, top, left and bottom, right). The peptide co-localizes with a staining with a conventional marker for the Golgi apparatus, BODIPY ceramide (red) (overlay yellow, bottom, right). This leads to the assumption that the specific peptide R-DIM-P-LF11-322 localizes to the Golgi within two hours, prior to induction of apoptosis.

#### Effect of peptides on plasma membranes and cholesterol distribution

**Fig 10:**
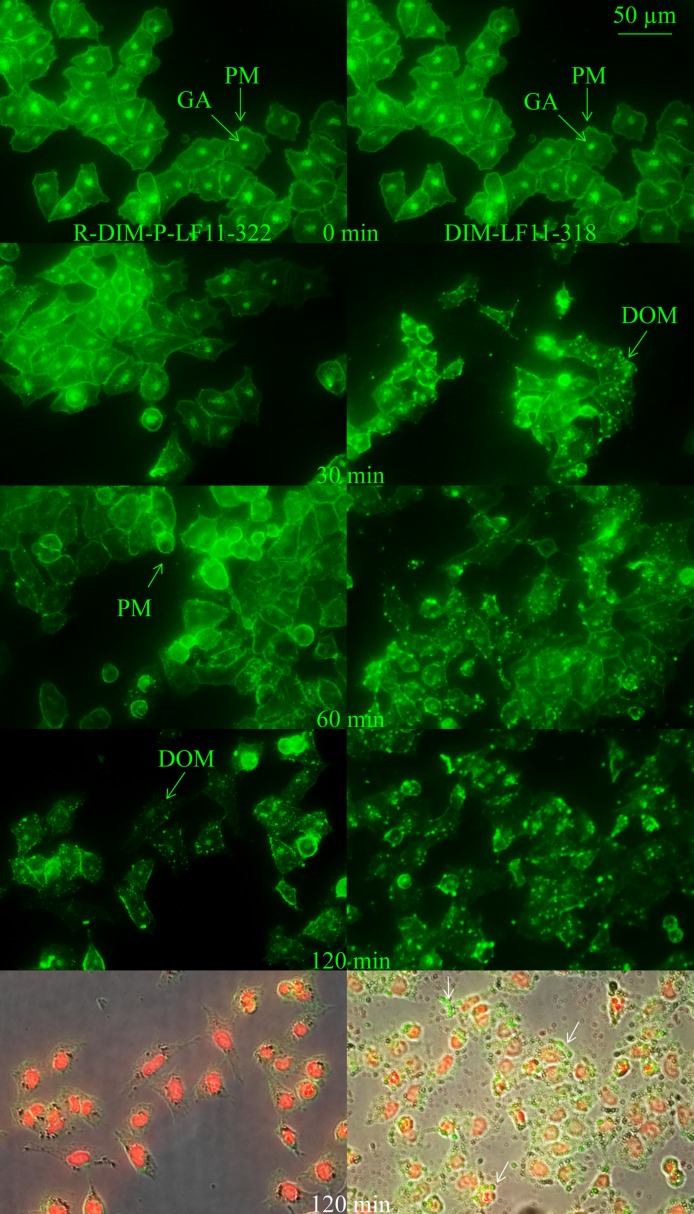
Changes in distribution of intracellular and plasma membrane cholesterol in cells of melanoma A375 upon peptide addition. A375 with stained cholesterol (filipin, green) were treated with 10 μM R-DIM-P-LF11-322 or DIM-LF11-318 for indicated time periods. Initial localization of cholesterol in PM and GA in untreated melanoma cells changes similarly, however with increased velocity upon treatment with DIM-LF11-318. Both peptides induce consumption of intracellular cholesterol and appearance of cholesterol rich domains in the plasma membrane after 30 min (DIM-LF11-318) or 60–120 min (R-DIM-P-LF11-322). Bottom OL BF: Overlay of bright field (OL BF) and fluorescence (green/filipin, staining of cholesterol; red/PI, staining of DNA of membrane damaged cells) upon 120 min of respective peptide incubation. DIM-LF11-318 causes release of small beads (white arrows), partially composed of cholesterol (green staining). Arrows indicate the plasma membrane (PM), Golgi apparatus (GA) as well as domains (DOM) and beads. Pictures are representative for a series of three experiments.

The first interaction point of the cationic peptides is the negatively charged PS exposed on the outer leaflet of the plasma membrane of cancer cells. To study possible differences in the effects of R-DIM-P-LF11-322 and DIM-LF11-318 on the plasma membrane and its cholesterol distribution, melanoma cells were incubated for 30 minutes up to two hours with respective peptides with subsequent staining of cholesterol. In absence of the peptide, cholesterol was found homogeneously distributed all over the plasma membrane (PM) as well as intracellularly, presumably in the Golgi (GA), as indicated by arrows (see Fig 10, Fig 9). Upon incubation with the two peptides similar changes on the plasma membrane, as well on cholesterol distribution were observed, though with different velocity and consequences for the plasma membrane. After 30 min DIM-LF11-318 and after 120 min R-DIM-P-LF11-322 caused total consumption of intracellular cholesterol, simultaneously the homogeneous distribution in the plasma membrane changed in formation of (punctual) domains (DOM). Whereas the PM of R-DIM-P-LF11-322 treated cells stayed intact over two hours, DIM-LF11-318 induced severe damage of the PM and dramatic release of small beads (white arrows) that were partially composed of cholesterol (green). Summarized, besides DIM-LF11-318 also R-DIM-P-LF11-322 shows induction of cholesterol domains in the plasma membrane and consumption or transport of intracellular cholesterol thereto, however, only DIM-LF11-318 induces visible lysis of the plasma membrane. The effects might indicate different stress responses of the cells to the presence of the peptides.

#### Effect of peptides on mitochondria

**Fig 11:**
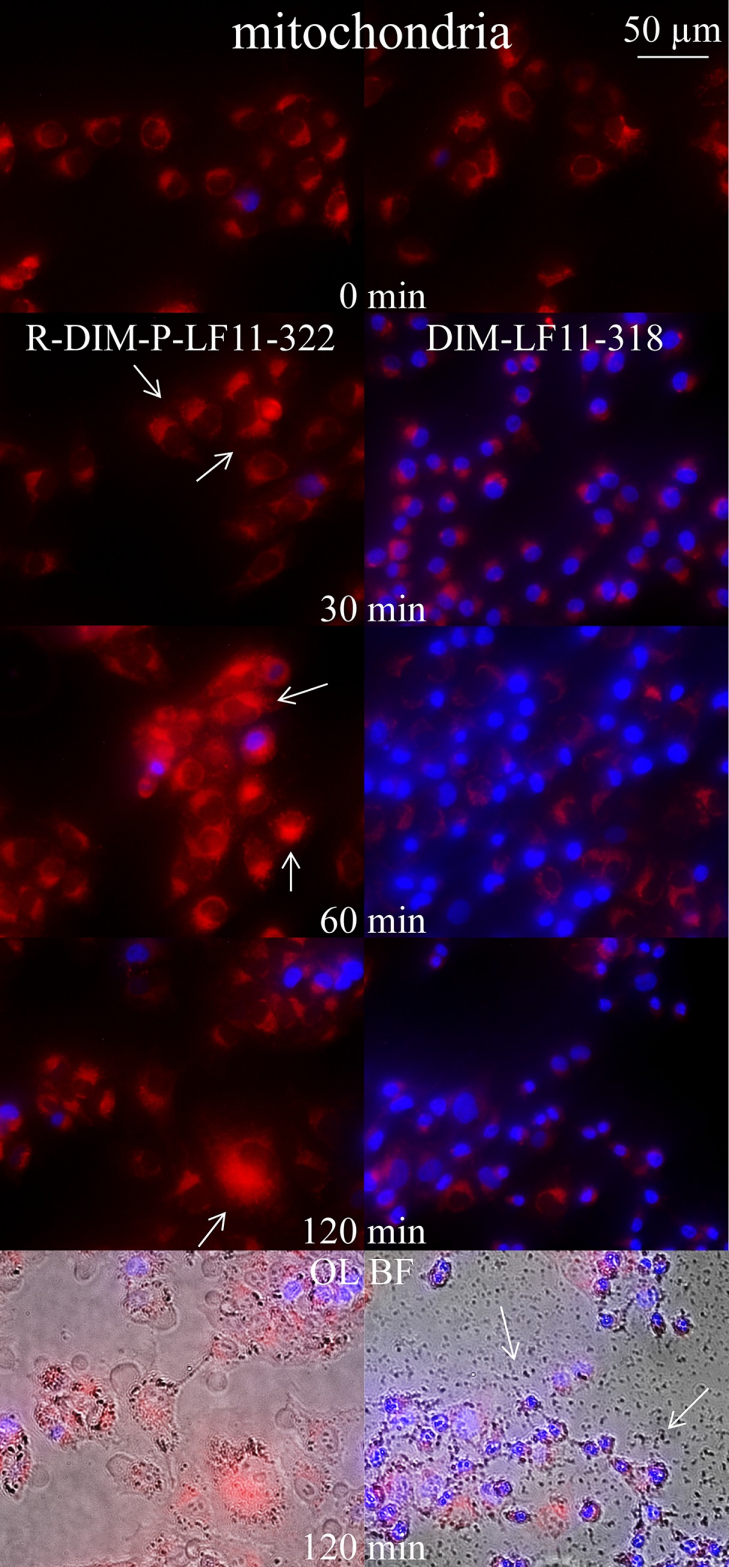
Changes in morphology of mitochondria of A375 upon incubation with R-DIM-P-LF11-322 and DIM-LF11-318. Overlay images of melanoma cells (A375) with stained mitochondria (red, MitoTracker Deep Red) and nucleus (blue, NucBlue) in presence of 10 μM peptide R-DIM-P-LF11-322 (left) or DIM-LF11-318 (right) at different incubation periods of 0, 30, 60 and 120 min. R-DIM-P-LF11-322 causes significant mitochondrial swelling (arrows), whereas DIM-LF11-318 mainly induces cell death indicated by increased uptake of nuclear staining with no significant swelling of mitochondria. Bottom OL BF: Overlay of bright field (OL BF) and respective fluorescence. Arrows indicate membrane debris upon incubation with DIM-LF11-318. Pictures are representative for a series of three experiments.

To study potential effects of R-DIM-P-LF11-322 or DIM-LF11-318 on mitochondria of melanoma cells (A375), they were incubated for 30 minutes up to two hours with the peptides followed by mitochondrial staining (Fig 11, left, without peptide). The effects were found to be severely different. R-DIM-P-LF11-322 induced significant swelling of mitochondria within two hours of incubation (Fig 11 right side, red staining). The majority of cells were “still” alive, indicated by absence of nuclear staining (blue) mainly occurring in dying cells. Such mitochondrial swelling is a sign of ongoing apoptosis. DIM-LF11-318 (right side) already induced cell death after 30 minutes, indicated by nuclear staining of the majority of cells and no significant alteration of the morphology of mitochondria within two hours. Further strong membrane lysis and release of cell debris was shown (Fig 11, OL BF; white arrows). This is consistent with published data by Riedl et al. [30], who reported that R-DIM-P-LF11-322 leads to apoptosis after 2 to 8 hours, whereas DIM-LF11-318 rapidly leads to necrosis of cancer cells and lysis of the plasma membrane within time periods of 30 minutes to two hours.

#### Effects of peptides on Golgi apparatus and ceramide distribution

**Fig 12:**
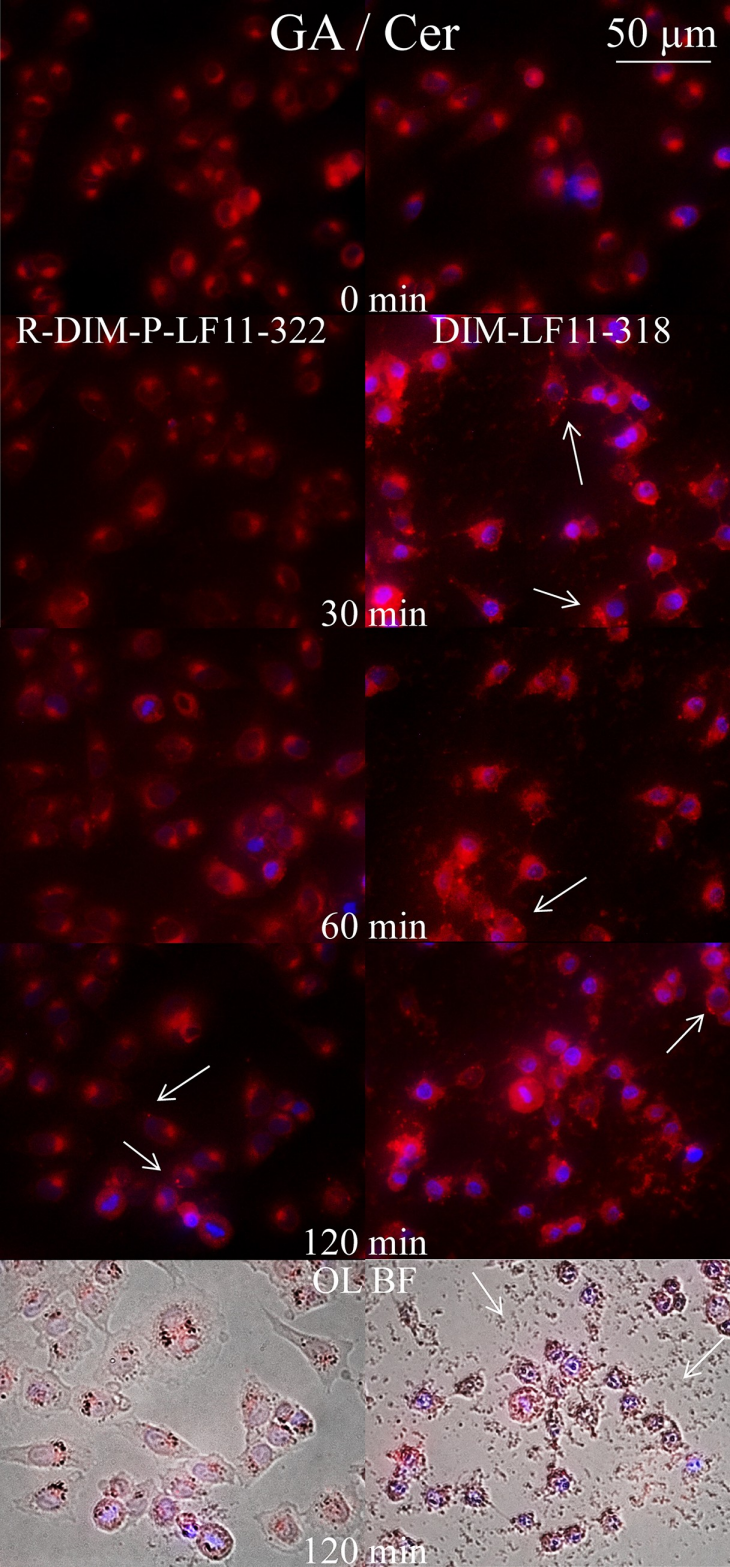
Changes in morphology of Golgi apparatus (GA) and distribution of ceramide (Cer) in A375 upon incubation with R-DIM-P-LF11-322 and DIM-LF11-318. Overlay images of melanoma cells (A375) with stained Golgi apparatus (GA), respectively ceramide (red, BODIPYceramide) and nucleus (blue, NucBlue R37606) in presence of 10 μM peptide R-DIM-P-LF11-322 (left) or DIM-LF11-318 (right) at different incubation periods of 0, 30, 60 and 120 min. R-DIM-P-LF11-322 causes distribution of the Golgi marker BODIPY ceramide into the cytosol and slight formation of domains in the plasma membrane (arrows) within 120 min indicating a strong effect on the Golgi apparatus and the distribution of ceramide. DIM-LF11-318 already induces strong staining of the cytosol and plasma membrane after 30 min, indicating even higher stress on the Golgi and formation of punctual domains of ceramide (arrows) in the plasma membrane. Bottom OL BF: Overlay of bright field (OL BF) and respective fluorescence. Arrows indicate membrane debris upon incubation with DIM-LF11-318. Pictures are representative for a series of three experiments.

To study potential differences in effects of peptides on another organelle involved in stress related response, the Golgi, as well as the so called “tumor suppressor” lipid ceramide, melanoma cells were incubated for 30 minutes up to two hours with R-DIM-P-LF11-322 and DIM-LF11-318, respectively. BODIPY ceramide was then added as vital stain for the Golgi apparatus, respectively detection of lipid trafficking of ceramide and metabolites. Ceramide is discussed as one of the mediators of cell death [56]. Interestingly R-DIM-P-LF11-322 obviously caused an altered distribution of the ceramide stain from the Golgi (0 min) to the cytosol or other organelles upon 60–120 minutes of peptide incubation (Fig 12 (left)) indicating a significant effect on the Golgi and lipid trafficking of ceramide or its metabolites. After 120 minutes domains of ceramides and/or metabolites appear in the plasma membrane (Fig 12, arrows), which is possibly involved in induction of cell death. This effect is dramatically increased in cells upon influence of DIM-LF11-318, where after 30 minutes the ceramide is dislocated from the Golgi to cytosol and plasma membrane in all cells, where a significant increase of ceramide derived from the Golgi appears concentrated in domains (Fig 12, right, arrows). Similar as shown for the peptide effect on the distribution of cholesterol, ceramide and/or metabolites seem to be transported to the plasma membrane in response to severe stress induced by the two peptides. Again as shown by the mitochondrial staining, R-DIM-P-LF11-322 acts more slowly and does not induce lysis of the plasma membrane, whereas DIM-LF11-318 causes more severe and rapid effects with signs of necrosis such as membrane lysis and release of cell debris (Fig 12, overlay with bright field, bottom). Ceramide which is discussed to be an inducer of cell death, when appearing in the PM, is in some cancer types reported to be reduced therein. Thus peptide induced transport of ceramide to the plasma membrane can be involved in initiation of tumor cell death.

For a better overview of obtained results see summary Table 4.

**Table 4:** Summary of characteristics and effects induced by R-DIM-P-LF11-322 and DIM-LF11-318 in model as well as in *in vitro* systems.

	R-DIM-P-LF11-322		DIM-LF11-318	
**sequence**	PFWRIRIRR- P -RRIRIRWFP-NH_2_		FWQRRIRRWRR-FWQRRIRRWRR-NH_2_	
**net charge**	+ 9		+ 13	
structure (PEPfold*)	β-sheet (loop)		α-helix (linear)	
	**model studies**			
**bilayer-perturbation (MLVs, OLVs) (DSC)**				
*non cancer mimic*	-		-	
*cancer mimic*	+++		++++	
*cancer mimic + Chol*	++		+++++	
**changes in lateral lipid distribution (GUVs)**				
*cancer mimic*	+++		++++	
**electrostatic & hydrophobic interaction (ULVs) (Zeta potential)**				
*non cancer mimic*	-		-	
*cancer mimic*	++ (el)		+++ (el + hy)	
*cancer mimic + Chol*	+ (el)		+++ (el + hy)	
	***in vitro* studies**			
**toxicity LC**_**50**_ **[**μ**M]**	4.2 ± 0.2 / 63.5 ± 1.3		3.8** ± 0.5 / 2.2** ± 0.9	
A375 / NHDF	cancer specific		active; non-specific	
**PS (PSD)**	dependence of activity		independence	
**Chol (MβCD)**	dependence of activity		independence	
**morphological changes lipid trafficking**	30 min	120 min	30 min	120 min
*mitochondria*	swelling	swelling	no swelling	no swelling
*Golgi apparatus / Cer*	~	stress response	stress response	stress response
		Cer trafficking	Cer trafficking	Cer trafficking
*plasma membrane / Chol*	~	domains	domains	domains + lysis
		Chol trafficking	Chol trafficking	Chol trafficking

Chol Cholesterol; el electrostatic; hy hydrophobic; Cer Ceramide; PSD PS-decarboxylase; MβCD methyl-β-cyclodextrin

(*) [33]

(**) [30]

(+) peptide effect, (++) stronger peptide effect etc.; (-) no peptide effect; (~) no influence.

## Discussion

In the last years it has been reported that cancer cells specifically expose the negatively charged lipid phosphatidylserine (PS) in the outer leaflet of the plasma membrane [7,9,10,15]. Thus, PS may provide a novel target for potential anticancer drugs. Accordingly, our lab revealed that specially refined derivatives of human lactoferricin (hLFcin) can specifically target the negatively charged PS [28]. We found that one LFcin derivative, R-DIM-P-LF11-322, interacts specifically with cancer cells, whereas another derivative DIM-LF11-318 also harms non-cancer cells [30]. Thus, the aim of this study was to reveal differences in mode of action and specificity of these two peptides using studies with model as well as *in vitro* systems, whereby the focus was on a tumor entity with poor prognosis, the malignant melanoma. Our results should help to further optimize the peptides in order to design a new, specific and potent therapy against this cancer type.

Since cancer cells specifically expose PS on the outer leaflet, cancer mimicking model systems composed of simply PS or mixtures with PC were used. Healthy cell mimics were composed of PC. Furthermore, cholesterol was added to the cancer model systems to determine if it affects the effectiveness, mode of action or specificity of the peptides, since cholesterol is present in high amounts in cancer as well as in non-cancer cell plasma membranes. In that respect, it was reported that cholesterol reduces the effectiveness of magainin, a host defense peptide of *Xenopus laevis*, on erythrocytes, and was therefore thought to exert a potential protective function against auto reactions against the host itself by its own defense peptides [50].

In cancer model systems where PS was present, R-DIM-P-LF11-322 showed electrostatic interactions (zeta potential) as well as perturbation of liposomes (DSC). All model studies with PS/PC mixtures revealed a specific interaction of R-DIM-P-LF11-322 with the cancer marker PS. DIM-LF11-318, however, exhibited a much stronger effect against the cancer model systems. It induced stronger perturbation of the cancer mimic and interestingly in presence of PS/PC mixtures effects on both components, PS and PC. Furthermore, zeta potential measurements in presence of DIM-LF11-318 showed hydrophobic besides electrostatic interactions. This ability to interact with anionic as well as neutral membrane surfaces is often related to low specificity *in vitro* [25]. A specific dependence of the antitumor activity of the cationic R-DIM-P-LF11-322 on the anionic cancer target PS was further confirmed with the enzyme PS-decarboxylase (PkPSD) that is able to convert (only) exposed (anionic) PS to neutral phosphatidylethanolamine (PE). By treatment with PSD, PS-exposure of the melanoma cells was reduced by significant amounts, which in consequence also significantly reduced the activity of the PS-specific peptide. This dependence on the target PS is in good correlation with previous reports [28,30] and presumably yields an important characteristic of the peptide for a cancer specific interaction. The non-specific DIM-LF11-318 differed markedly by showing toxicity on cancer cells not in need for exposed PS, which might explain its non-selective activity on PS-exposing cancer as well as non-exposing non-malignant cells.

No effects according bilayer perturbation (DSC), electrostatic (zeta potential) or hydrophobic interactions (zeta potential) were seen for non-cancer mimics (pure DPPC) for R-DIM-P-LF11-322. This is in agreement with its non-toxicity on healthy cells and studies performed by Riedl et al. [30] showing that R-DIM-P-LF11-322 only affects model systems, when PS is present. However, it is of interest that DIM-LF11-318 did only exhibit low effects on healthy cell mimicking model systems, although it leads to strong cell death of normal human dermal fibroblasts or melanocytes [30]. Possible explanation is given in previous work [30], where it has been shown that DIM-LF11-318 can penetrate POPC bilayers without causing severe membrane perturbation, comparable to cell penetrating peptides [57,58]. Killing of non-cancer cells by DIM-LF11-318 was thus suggested to occur after passage through the plasma membranes by subsequent cell lysis from the inside of the plasma membrane, where PS is present, rather than from the outside [30]. Model studies though revealed that DIM-LF11-318 has an effect on DPPC when DPPS was present.

Interestingly addition of cholesterol to PC/PS model systems revealed a decrease in the efficacy of R-DIM-P-LF11-322 against the cancer mimics. One would assume that the presence of cholesterol increases the fluidity and therefore the peptide susceptibility of the membrane. Indeed cholesterol increased the effect of the non-specific DIM-LF11-318. DIM-LF11-318 was shown to interact not only with PS but also with PC, an effect even enhanced through presence of cholesterol. Zeta potential experiments showed that the first interaction of R-DIM-P-LF11-322 with the target membrane occurs via electrostatic interactions, whereas increasing amounts of cholesterol seemed to lead to a decrease in electrostatic interactions between the lipid model system and the peptide. On the contrary, DIM-LF11-318 as discussed seems to interact via electrostatic as well as hydrophobic forces, whereas however increasing amounts of cholesterol had no influence on the peptide effect on the zeta potential. The data with lipid model systems are in perfect correlation with zeta potential changes determined with cancer and non-cancer cells in presence of both peptides, confirming the main interaction of the peptides with lipid targets [30]. The interaction of DIM-LF11-318 with the cancer target PS, but also with PC, even enhanced in the presence of cholesterol, the latter two present in cancer as well as healthy cells, suggests that these interactions are driving the non-specificity of the peptide. The interaction of the cancer specific R-DIM-P-LF11-322, however, seems to be exclusively triggered by the presence of PS specifically exposed on the cell surface of cancer cells.

**Fig 13:**
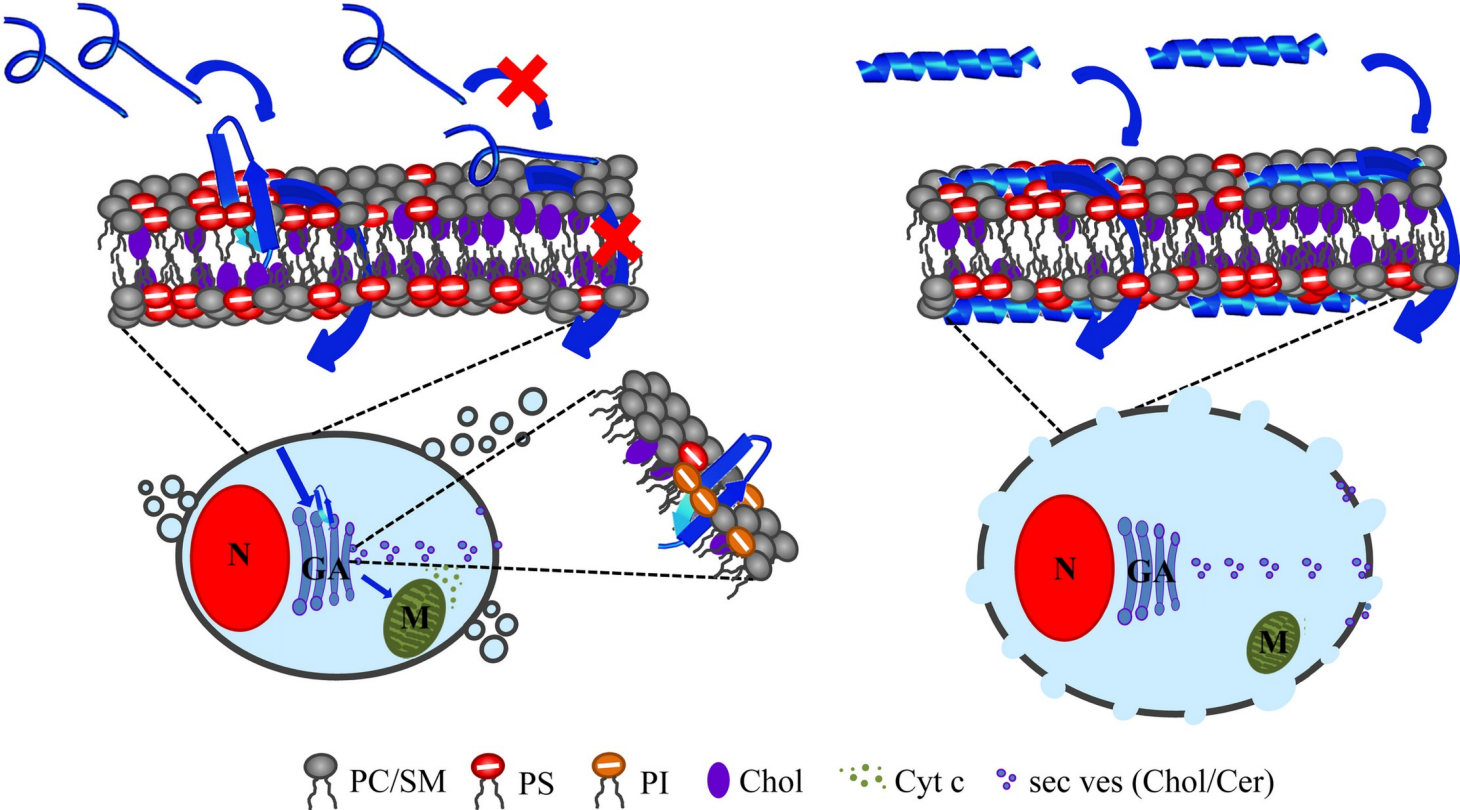
Predicted modes of action of R-DIM-P-LF11-322 and DIM-LF11-318 on cancer cells. Cancer cells specifically expose the negatively charged lipid phosphatidylserine (PS, red) in the outer leaflet of the plasma membrane [7,15] offering a target for the cationic amphipathic peptides. Cholesterol (Chol, purple) is enriched in so called rafts together with phosphatidylcholine and sphingomyelin (PC and SM, gray) [65]. Both peptides cause severe changes in lipid distribution, leading to defect borderlines and via primary and secondary effects to different ways of cell death. Left: R-DIM-P-LF11-322 (blue) forms its membrane active structure (β-sheet) only in presence of PS [28,30], which however can be partially hindered by the presence of cholesterol. At PS enriched sites, though, R-DIM-P-LF11-322 with its active structure can successfully enter the cell and reach an intracellular target, presumably the Golgi apparatus (GA) harboring negatively charged lipids as PS or phosphatidylinositol (PI, orange) leading to lipid trafficking, domain formation in the plasma membrane and mitochondrial swelling. Apoptosis occurs via different ways, e.g., by inducing loss of membrane potential of mitochondria (M) which leads to cytochrome c (Cyt c) efflux and transport of secretory vesicles (sec ves) carrying Chol and ceramide (Cer). Right: DIM-LF11-318 (blue) forms its membrane active structure (α-helix) in presence of both, PS and PC, independently of cholesterol. DIM-LF11-318 is thought to harm the cell rapidly and directly at the plasma membrane leading to necrosis indicated by membrane lysis and release of cell debris. Intracellularly, strong stress is induced shown by lipid trafficking to and changes in domains of the plasma membrane. Modified from Riedl et al. [30].

Importantly, this different effect of cholesterol on R-DIM-P-LF11-322 and DIM-LF11-318 in the model studies was also confirmed *in vitro*. Melanoma cells were therefor treated with an agent depleting cholesterol of the plasma membrane. In accordance to the model studies cholesterol depletion led to an increase of the killing efficiency of R-DIM-P-LF11-322, while no influence was seen for DIM-LF11-318. An effect of cholesterol decreasing the activity of certain (specific) host defense peptides has already been described by Matsuzaki et al. [50]. They reported that upon cholesterol depletion of erythrocytes, the effect of the antimicrobial and cancer specific peptide magainin (frog skin) increases, indicating that cholesterol is able to reduce activity of certain peptides. This may be a way for host cells to protect themselves against membrane disrupting effects by its own host defense. This is even more reasonable when thinking of the main role of host defense peptides, the protection of the host from (pathogenic) bacteria, which are with very rare exceptions devoid of (membrane) cholesterol. A reduced disrupting effect in presence of cholesterol was also described for other AMPs as gramicidin S [59], MSI variants [60] or latarcin 2a [61]. Interestingly, Matsuzaki et al. [50] also found that the non-specific peptide melittin (bee) is similar to the non-specific DIM-LF11-318 not being influenced by cholesterol depletion, and exhibits the same effects independent of cholesterol content in erythrocytes. Therefore, the dependence of sensitivity on cholesterol is seemingly playing an important role on peptide specificity as also revealed in our studies. Two explanations have been discussed so far. First, cholesterol is responsible for stiffening the membrane and therefore decreasing the lytic activity of the peptides [62]. Second, in 1999 Matsuzaki et al. [63] suggested another reason for an activity decreasing effect of cholesterol on specific peptides, which may occur by prevention of the formation of a membrane active secondary structure of the peptide when binding to the phospholipid bilayer. By formation of a hydrogen bond between the hydroxyl group of cholesterol and the peptide molecule, it might inhibit intramolecular hydrogen bond formation within the peptide which is probably needed for stability and its effectivity on membranes. In contrast, the non-specific melittin configures already a well-defined structure in aqueous solution in absence of the target prone to interact with all membranes, thus cholesterol cannot exert its protective role any more. R-DIM-P-LF11-322, on the contrary has been reported to adopt a β-sheet structure only in presence of an anionic lipid environment (cancer membrane) [30]. Therefore, cholesterol may prevent the formation of its active secondary structure, consequently inhibiting its toxicity on normal cells. This assumption is supported by CD-experiments performed with liposomes composed of PS and PS/cholesterol, where the adoption of a β-sheet structure in presence of the anionic lipid environment was strongly reduced by the addition of cholesterol (suppl., S2 Fig). However, peptide activity is not completely reduced when cholesterol is present, if, as in the case of cancer membranes, also the target PS is present. Cholesterol and non-exposure of PS thus might be the clue for the protection from toxicity on normal cells of the host. Accordingly DIM-LF11-318 was shown to develop an α-helical structure in presence of the cancer and the non-cancer mimic acting rapidly [30]. Thus, a fast and non-specific formation of an active peptide structure might not be prevented by cholesterol and lead to non-specific interactions (for a schematic depiction see Fig 13).

The plasma membrane of mammalian cells is reported to comprise certain micro-domains with increased levels of protein receptors and lipids as PC, SM and cholesterol, termed lipid rafts. Rietveld et al. [64] assigned these lipid rafts in model membranes to an immiscibility of ordered (L_o_ phase) and disordered (L_d_ or L_α_ phase) liquid phases. For some cancer types the amount, for others the distribution of cholesterol is discussed to be altered in their plasma membranes [17,65]. Besides, for cancer cells changes in these micro-domains have been discussed, thus Li et al. [65] stated that some cancer types like breast or prostate cancer exhibit elevated levels of cholesterol-enriched lipid rafts, whereas the rest of the membrane contains less cholesterol. Presumably these regions with reduced cholesterol exhibiting increased fluidity are also enriched with PS. AMP derived anti-cancer peptides may better reach their target PS, form their active structure mainly unaffected by cholesterol and further the decreased stiffness may support incorporation of the peptides [17]. Since non-cancer membranes have a higher content of cholesterol in non-raft regions and therefore are more rigid and they lack anionic lipids in the outer leaflet of the membrane, specific anti-cancer peptides may not form their active structure and harm them [17]. This leads to the assumption that the stiffness of healthy cell membranes and the absence of PS are major reasons why the cells are protected against specific host defense peptides while it has no influence on already structured non-specific peptides.

To study a possible impact of naturally occurring domains experiments were performed using mimics with an inhomogeneous distribution of lipids. Both peptides within this study acted as well or even better (R-DIM-P-LF11-322) when domains were present, assuring activity of peptides not only on artificial model systems, but even on natural cancer cell membranes *in vitro* and *in vivo*. Studies with GUVs revealed that upon peptide addition a dramatic change in the distribution of lipid domains occurred. Bright spots indicated formation of peptide induced and enriched macroscopic regions of disordered lipids, presumably PS. This is conform with reports of Epand et al. [66] that arginine rich peptides (like LF11-322; there named PR-9) induce segregation of anionic and zwitterionic lipids. This clustering of lipids generates defects between peptide poor and peptide rich domains which may lead to membrane permeabilization and secondary effects as changes of protein activity [66,67]. Measurements of GUVs composed of PC/PS and cholesterol further revealed that the size of the particles increases upon addition of both peptides; often also adhesion of multiple GUVs was observed, a possible cross-linking by the peptides interacting with PS cannot be excluded. Such effects have also been shown for other amphipathic peptides as RW16 described by interacting like a glue between anionic vesicles [68]. *In vitro* and *in vivo* this cross-linking could lead to network of cancer cells, severely disturbed in their normal signaling and cell contact. Phase separation was also shown in DSC studies upon addition of R-DIM-P-LF11-322 to DPPS liposomes and PS/PC mixtures in presence of cholesterol.

Changes in the lipid (cholesterol) distribution and formation of domains in the plasma membrane were further seen *in vitro* when melanoma cells were incubated with peptide. The addition of the non-specific DIM-LF11-318 rapidly resulted in a strong change in cholesterol distribution in the plasma membrane. As well, intracellular cholesterol was consumed indicating induction of strong cellular stress. The same phenomena were observed in presence of R-DIM-P-LF11-322, though appearing more slowly.

To study further different effects induced by the two peptides *in vitro* morphological changes of mitochondria and Golgi upon peptide addition were studied within this work.

In a previous study Riedl et al. have already shown that R-DIM-P-LF11-322 interacts by (slow) induction of apoptosis in the cancer cell *in vitro* [30], therefor the peptide has to enter the cell and reach an inner target to provoke the intrinsic pathway of the program cell death.

Indeed, localization studies revealed that after 2 hours the fluorescently labeled R-DIM-P-LF11-322 mainly localized to the Golgi. R-DIM-P-LF11-322 may enter the cell in a manner analogous to the so-called sinking raft model where the peptide localizes at the membrane surface. Only in the presence of exposed PS and at a certain amount it sinks into the bilayer [69] and then localizes to the Golgi by interaction with negatively charged lipids, as PS or phosphatidylinositol (PI) (see Fig 13 model). Experiments with an inhibitor of clathrin and caveolae mediated endocytosis (MβCD) exclude these routes of cell entrance. The need for μM amounts further excludes peptide interaction via pore formation or receptor mediated uptake. Localization to Golgi was at the first moment surprising, since the main starting point of apoptosis is often described to be the mitochondria [70]. However, such localization of potential anti-cancer drugs to Golgi has also been reported before, e.g., for the Shiga-Toxin B subunit [71]. Further, it was reported that the Golgi upon stress sensing can activate pro-survival mechanisms as well as suicide programs if stress results in irreparable damage [54,72]. This may be due to the activation of caspase-2 which is besides other regions also located in the Golgi. Caspase-2 cleaves golgin160 which was reported to be an early event of apoptosis [73,74]. Indeed, despite localization to Golgi R-DIM-P-LF11-322 also showed effect on mitochondria by induction of mitochondrial swelling being a sign of ongoing apoptosis, which is as discussed in accordance with previous reports that upon incubation with R-DIM-P-LF11-322 the caspase-3 and -7 activities increase [30]. Since the peptide was shown to localize to the Golgi apparatus we also had a closer look on the organelle morphology upon peptide incubation. And indeed the peptide caused severe changes in the distribution of a ceramide label used as vital Golgi stain. The ceramide and/or metabolites were dislocated from the organelle to the cytosol, other organelles and plasma membrane, being a strong indication of stress response of the cancer cell upon peptide treatment. Appearance of the rigid ceramide or metabolites upon peptide treatment in the normally fluid plasma membrane can play strong primary and secondary effects on membrane integrity by changes in fluidity, permeability or activity of proteins important for vitality. Indeed, one way for activation of apoptosis by the Golgi was reported to be the conversion of ceramide into the ganglioside GD3 by GD3 synthase. GD3 is then transported to the mitochondrion provoking mitochondrial membrane permeability [75,76] which then leads to induction of the intrinsic pathway of apoptosis via cytochrome c efflux. Furthermore, another link between Golgi and apoptosis is suggested to be the transport of death receptors (like Fas or TNF-receptor 1) upon stress signaling to the plasma membrane since death receptors are localized under non-stress conditions in the Golgi [72,74,77]. This may also be the reason why intracellular cholesterol of the Golgi was seen to disappear upon peptide incubation, since the transport of the death receptors occurs via vesicle transport where cholesterol would be located in the vesicle membrane.

DIM-LF11-318, which is known to act rapidly via membrane lysis [30], showed similar changes on the distribution of the ceramide stain from the Golgi to cytosol and plasma membrane, however much faster and accompanied by early start of cell death and severe lysis and distortion of the membrane. Also the depletion of intracellular cholesterol and formation of domains within the plasma membrane occurred much faster and earlier again together with signs of membrane lysis and cell death. No swelling of the mitochondria was observed. The results confirmed once more that DIM-LF11-318 acts more strongly and faster and leads to necrosis rather than apoptosis which is in agreement with Riedl et al. [30].

Both peptides induce severe stress on cancer cells even though different killing mechanisms seem to be induced before cell death. The potential role of ceramide in killing by both peptides is very interesting, since ceramide belongs to the so-called tumor suppressor lipids and an inhibition of its formation is reported to be involved in tumor progression as well as resistance to cytostatic drugs. Anticancer drugs that can (re-) induce synthesis of ceramide or transport to the plasma membrane are then able to trigger tumor suppressive and anti-proliferative effects such as apoptosis, autophagy, senescence, and necroptosis [78].

## Conclusion

Within this work, it was shown that the cancer specific peptide R-DIM-P-LF11-322 and the non-specific DIM-LF11-318 alter the lipid distribution and fluidity of cancer cell membranes, which may lead to changes of the permeability of the plasma membrane and to secondary effects like loss of function of membrane proteins or release of stress signals (mechanistic scheme, see Fig 13). More importantly, we could prove that R-DIM-P-LF11-322 specifically interacts with PS exposed in the outer leaflet of cancer cell plasma membranes. It is less effective when cholesterol is present but still leads to specific death of cancer cells. Contrary, the non-specific DIM-LF11-318 preferentially interacts with PS but can also interact with neutral lipids like PC. The non-specific peptide is unaffected by cholesterol. This different ability of lipid discrimination and sensibility to cholesterol might already indicate one crucial feature for a cancer specific peptide such as R-DIM-P-LF11-322. One conceivable reason may be that cholesterol can prevent the formation of the membrane active β-sheet structure of the specific peptide. Furthermore, it was shown that R-DIM-P-LF11-322 specifically enters the cancer cell through the plasma membrane via PS and then localizes to the Golgi, thereby constituting a stress signal which leads to several effects as changes of Golgi composition, cholesterol and ceramide transport to the plasma membrane, domain formation therein, swelling of mitochondria and further induction of apoptosis (in confirmation with Riedl et al. [30]). In contrast, DIM-LF11-318 seems to interact non-specifically with PS and PC on the plasma membrane independently on cholesterol, adopting its membrane active α-helical structure, where it immediately leads to membrane lysis with no need of an intracellular target. In summary, the results reveal that both peptides kill melanoma cells after strong induction of cellular stress, however, the different mode and site of interaction decide on their specificity.

## Supporting information

S1 Fig Stable depletion of cholesterol of plasma membrane (PM) by MβCD over two hours of recovery. Cells (A375) were treated with 0 (left) or 5 mM MβCD (right) in DBPS supplemented with 10% FBS with gentle shaking at 100 rpm and 37°C. Top: Filipin staining shows cholesterol localization without MβCD treatment in Golgi and plasma membrane (left) but cholesterol depletion of plasma membrane upon MβCD treatment (right). Bottom: Solution was removed and DMEM supplemented with 10% FBS was added. Recovery of two hours was allowed. It reveals a stable depletion of plasma membrane cholesterol by MβCD, seen by prevention of re-transport of cholesterol into plasma membrane over two hours (lower right).
(TIF)

S2 Fig Effect of cholesterol on secondary structure of R-DIM-P-LF11-322. Secondary structures of R-DIM-P-LF11-322 in Hepes buffer (black lines) or presence of POPS (gray lines) and POPS/Cholesterol (3:1; molar ratio) (light gray lines) at peptide to surfactant ratios of 1:25 were calculated (see inset) from respective CD spectra. Inset: The analyzed α-helical content is shown in black at the bottom, β-turns are demonstrated in light grey, turns in dark grey and random coil structures in white at the top. Analyzed proportions, given in the columns as percentages, were calculated using the Dichroweb, Contin_LL (Provencher & Glockner Method) Convolution Program (see Methods). Specific peptide R-DIM-P-LF11-322 changes its secondary structure only in the presence of the cancer mimic POPS. Cholesterol can strongly reduce such a change in conformation and thereby reduce the peptide activity.
(TIF)

S1 Table Zeta potential and size. Values of DPPC, DPPS or DPPC/DPPS/Cholesterol (1:1:0, 1:1:0.25 and 1:1:0.5; molar ratios) liposomes in absence and presence of R-DIM-P-LF11-322, DIM-LF11-318 (lipid to peptide molar ratio) or CaCl_2_ (1mM). (See also Figs 2 and 6). Data analysis was processed using the instrumental Malvern’s DTS software. Mean Zeta-potential and size value are calculated from the means of 30 runs of three measurements of three independent experimental repetitions.
(DOCX)
