# Supplementary figures and images for: Interaction of two antitumor peptides with membrane lipids – Influence of phosphatidylserine and cholesterol on specificity for melanoma cells
Source: PLoS One. 2019 Jan 25;14(1):e0211187. doi: 10.1371/journal.pone.0211187 (PMC6347193; doi:10.1371/journal.pone.0211187)

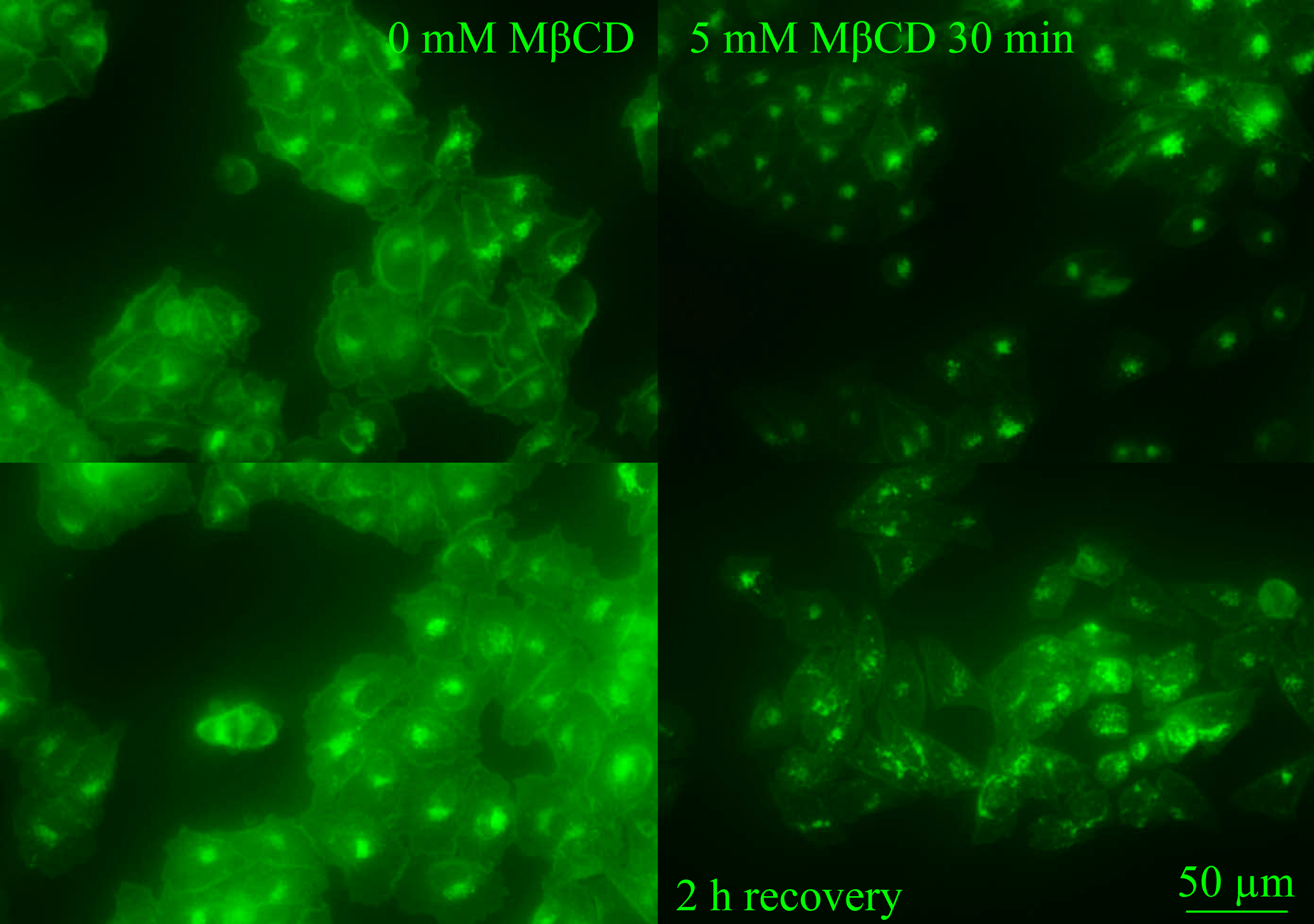

Supplement: S1 Fig — Cells (A375) were treated with 0 (left) or 5 mM MβCD (right) in DBPS supplemented with 10% FBS with gentle shaking at 100 rpm and 37°C. Top: Filipin staining shows cholesterol localization without MβCD treatment in Golgi and plasma membrane (left) but cholesterol depletion of plasma membrane upon MβCD treatment (right). Bottom: Solution was removed and DMEM supplemented with 10% FBS was added. Recovery of two hours was allowed. It reveals a stable depletion of plasma membrane cholesterol by MβCD, seen by prevention of re-transport of cholesterol into plasma membrane over two hours (lower right). (TIF) [file pone.0211187.s001.tif]
