# Supplementary material for: Interaction of two antitumor peptides with membrane lipids – Influence of phosphatidylserine and cholesterol on specificity for melanoma cells
Source: PLoS One. 2019 Jan 25;14(1):e0211187. doi: 10.1371/journal.pone.0211187 (PMC6347193; doi:10.1371/journal.pone.0211187)
Supplement: S1 Table — Values of DPPC, DPPS or DPPC/DPPS/Cholesterol (1:1:0, 1:1:0.25 and 1:1:0.5; molar ratios) liposomes in absence and presence of R-DIM-P-LF11-322, DIM-LF11-318 (lipid to peptide molar ratio) or CaCl2 (1mM). (See also Figs 2 and 6). Data analysis was processed using the instrumental Malvern’s DTS software. Mean Zeta-potential and size value are calculated from the means of 30 runs of three measurements of three independent experimental repetitions. (DOCX) [file pone.0211187.s003.docx]

**S1 Table: Zeta potential and size.** Values of DPPC, DPPS or DPPC/DPPS/Cholesterol (1:1:0, 1:1:0.25 and 1:1:0.5; molar ratios) liposomes in absence and presence of R-DIM-P-LF11-322, DIM-LF11-318 (lipid to peptide molar ratio) or CaCl_2_ (1mM). (See also Figs 2 and 6). Data analysis was processed using the instrumental Malvern’s DTS software. Mean Zeta-potential and size value are calculated from the means of 30 runs of three measurements of three independent experimental repetitions.

|  | **Zeta potential [mV]** | **Zeta**  **deviation**  **[mv]** | **Size (z-average)/ Number mean*[nm]** | **Polydispersity Index**  **(pdi)** |
| --- | --- | --- | --- | --- |
| **DPPC** | ­ + 3 ± 2 | 4 ± 1 | 210 ± 10 | 0.2 ± 0,1 |
| + R-DIM-P-LF11-322 | + 5 ± 3 | 5 ± 0 | 230 ± 30 | 0.3 ± 0.0 |
| + DIM-LF11-318 | + 6 ± 1 | 5 ± 0 | 160 ± 5 | 0.2 ± 0.0 |
| + CaCl_2_ | + 9 ± 4 | 5 ± 0 | 140 ± 30 | 0.3 ± 0.2 |
| **DPPS** | - 60 ± 4 | 11 ± 7 | 130 ± 5 | 0.3 ± 0.2 |
| + R-DIM-P-LF11-322 | - 4 ± 6 | 9 ± 1 | 1200 ± 150 | 0.5 ± 0.1 |
| + DIM-LF11-318 | + 18 ± 3 | 4 ± 0 | 1700 ± 550 | 0.5 ± 0.3 |
| + CaCl_2_ (1 mM) | - 22 ± 5 | 7 ± 1 | 160 ± 45 | 0.2 ± 0.1 |
| **DPPC/DPPS/Cholesterol (1:1:0)** | - 46 ± 15 | 17 ± 4 | 120 ± 10 | 0.2 ± 0.1 |
| + R-DIM-P-LF11-322 | + 5 ± 3 | 4 ± 2 | 900 ± 180 | 0.3 ± 0.1 |
| + DIM-LF11-318 | + 20 ± 2 | 4 ± 1 | 270 ± 60 /940 ± 260 | 0.3 ± 0.1 |
| + CaCl_2_ | - 16 ± 3 | 7 ± 1 | 90 ± 15 | 0.6 ± 0.1 |
| **DPPC/DPPS/Cholesterol (1:1:0.25)** | - 49 ± 4 | 12 ± 5 | 100 ± 10 | 0.1 ± 0.0 |
| + R-DIM-P-LF11-322 | - 3 ± 2 | 4 ± 1 | 1100 ± 500 | 0.4 ± 0.1 |
| + DIM-LF11-318 | + 22 ± 2 | 5 ± 0 | 840 ± 230 | 0.2 ± 0.1 |
| + CaCl_2_ (1 mM) | - 18 ± 2 | 5 ± 1 | 110 ± 5 | 0.2 ± 0.1 |
| **DPPC/DPPS/Cholesterol (1:1:0.5)** | - 61 ± 5 | 11 ± 3 | 130 ± 10.0 | 0.1 ± 0.0 |
| + R-DIM-P-LF11-322 | - 15 ± 8 | 3 ± 0 | 1100 ± 210 | 0.4 ± 0.2 |
| + DIM-LF11-318 | + 18 ± 2 | 5 ± 1 | 1260 ± 180 | 0.4 ± 0.2 |
| + CaCl_2_ | - 23 ± 0 | 7 ± 0 | 1. ± 5 | 0.1 ± 0.0 |

*Z-average was taken when polydispersity index (pdi) was below 0.5; number mean was taken when pdi was above 0.5
